# Structural basis of membrane machines that traffick and attach heme to cytochromes

**DOI:** 10.1016/j.jbc.2023.105332

**Published:** 2023-10-10

**Authors:** Jonathan Q. Huynh, Ethan P. Lowder, Robert G. Kranz

**Affiliations:** Department of Biology, Washington University, St. Louis, Missouri, USA

**Keywords:** heme, cytochrome c, structure, molecular machine, transport, heme attachment, cytochrome, leaflet, bioenergetics, biogenesis

## Abstract

We evaluate cryoEM and crystal structures of two molecular machines that traffick heme and attach it to cytochrome c (cyt c), the second activity performed by a cyt c synthase. These integral membrane proteins, CcsBA and CcmF/H, both covalently attach heme to cyt c, but carry it out *via* different mechanisms. A CcsB-CcsA complex transports heme through a channel to its external active site, where it forms two thioethers between reduced (Fe^+2^) heme and CysXxxXxxCysHis in cyt c. The active site is formed by a periplasmic WWD sequence and two histidines (P-His1 and P-His2). We evaluate each proposed functional domain in CcsBA cryoEM densities, exploring their presence in other CcsB-CcsA proteins from a wide distribution of organisms (*e.g.*, from Gram positive to Gram negative bacteria to chloroplasts.) Two conserved pockets, for the first and second cysteines of CXXCH, explain stereochemical heme attachment. In addition to other universal features, a conserved periplasmic beta stranded structure, called the beta cap, protects the active site when external heme is not present. Analysis of CcmF/H, here called an oxidoreductase and cyt c synthase, addresses mechanisms of heme access and attachment. We provide evidence that CcmF/H receives Fe^+3^ heme from holoCcmE *via* a periplasmic entry point in CcmF, whereby heme is inserted directly into a conserved WWD/P-His domain from above. Evidence suggests that CcmF acts as a heme reductase, reducing holoCcmE (to Fe^+2^) through a transmembrane electron transfer conduit, which initiates a complicated series of events at the active site.

Cytochromes are heme proteins involved in electron transport in the cell. Maturation of cytochromes often requires accessory factors for heme insertion and/or protein complex formation. The assembly of c-type cytochromes (cyt c) is complicated, in part because reduced heme (Fe^+2^) is posttranslationally attached to the apoprotein by a cyt c synthase (reviewed in (1, 2, 3, 4, 5)). The covalent attachment is *via* two thioether bonds between the two vinyl groups on heme to two cysteines of a CXXCH motif in cyt c. The cyt c apoprotein is unfolded prior to attachment so both the unfolded apoprotein and heme must be trafficked to the synthase active site. Attachment is stereospecific, the first cysteine of CXXCH is always to the 2-vinyl and the second cysteine is to the 4-vinyl groups of heme. Upon thioether formation, the holocyt c is released from the cyt c synthase, which is sometimes called a cyt c heme lyase (see below). Following release, the cyt c protein folds into its native state.

It has been known for over 2 decades that three pathways in nature exist for the biogenesis of c-type cytochromes, called systems I, II, and III (6). System III in mitochondria is a soluble enzyme (holocytochrome c synthase, HCCS) in the intermembrane space where cyt c and cyt c1 function (7). HCCS binds reduced heme and attaches it to the cysteines of the CXXCH motifs (8, 9, 10). The trafficking of heme in eukaryotes has been an active area of research (11, 12, 13, 14, 15, 16), but how heme moves to the intermembrane space is largely unknown. The mechanisms and outstanding questions underlying the system III HCCS have been reviewed recently (17).

This manuscript concerns the much older (evolutionarily) systems I and II (Fig. 1, *A* and *B*), which are more complicated than system III. System I and II are present in bacteria, archaea, and some mitochondria (Fig. 1*C*). Since all c-type cytochromes are in the periplasmic space, these pathways transport heme to outside the inner membrane and covalently attach it to the CXXCH motif (Fig. 1, *A* and *B*). All cyt c apoproteins possess a SEC-dependent signal sequence for secretion to the outside. Roles in heme transport and attachment for the eight proteins (CcmA-H) in System I, and two (CcsB and CcsA) in system II, all membrane proteins, have been suggested (*e.g.*, (1, 2, 3, 4, 5)). Particularly noteworthy in these systems are three protein subfamilies (CcmC, CcmF, and CcsA) that are structurally defined by a conserved tryptophan-rich domain (WWD or trp-trp-asp) (18), adjacent to two histidines facing the periplasmic space (called P-His1 and 2 here) (19). This WWD/P-His domain has been shown to bind heme, based on spectroscopic (1, 20), cross-linking (21, 22), and biochemical genetic studies. The family has been termed “heme handling proteins” (23). In the last 2 years, the three dimensional structures of all three family members have been determined (24, 25, 26), providing extraordinary molecular detail to understand heme trafficking and heme attachment (Fig. 2, *A*–*D*). Here, we explore the structures to examine the structural basis for mechanisms and universal features of key domains. We focus on the CcsBA and CcmF/H complexes, which have been termed multifunctional molecular machines (24, 25), machines that carry out complex heme trafficking and cyt c attachment roles (*i.e.*, cyt c synthases).

**Figure 1:**
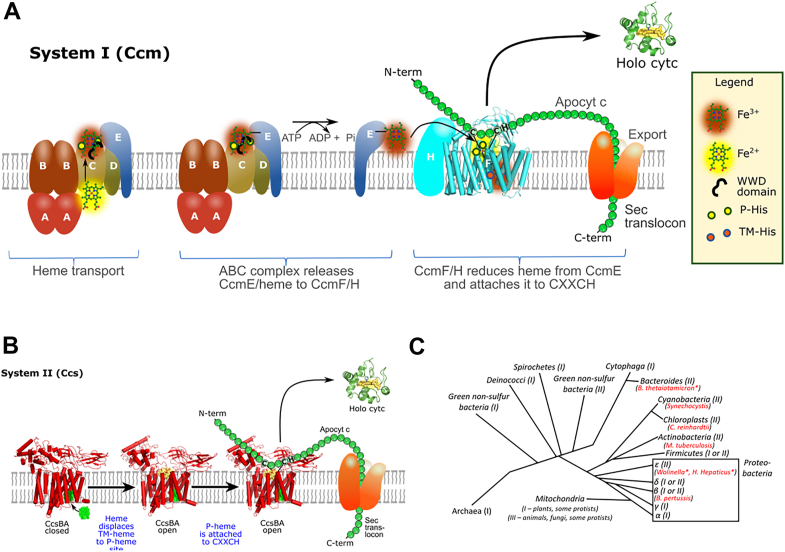
**The cyt c assembly pathways called System I and II.***A*, *cartoon* of the system I pathway encoded by eight *ccm* genes, displayed in two steps: the synthesis of holoCcmE and the use of heme from holoCcmE for assembly of cyt c. Proteins that reduce the thiols of the cyt c (CXXCH motif) before attachment (CcmG, DsbD) are not shown. *Cartoon* is used from (26). *B*, *cartoon* of system II showing the bifunctional CcsBA protein, a heme transporter and cyt c synthase. The model displays and derives from the cryoEM structures of CcsBA from *H. hepaticus*. Proteins that reduce the thiols of the cyt c (CXXCH motif) before attachment are not shown. *C*, evolutionary tree showing distribution of system I and II. The CcsB/A proteins (system II) from organisms listed in *red* are analyzed in this study, using a combination of computational structures (by RosseTTAfold) and fitted (with chimera) into cryo-EM densities of *H. hepaticus* CcsBA. Organisms labeled *red* with an *asterisk* (∗) have naturally fused CcsBA proteins while the others have separate *ccsB* and *ccsA* genes (unfused CcsB-CcsA complexes). The tree is modified from (37).

**Figure 2:**
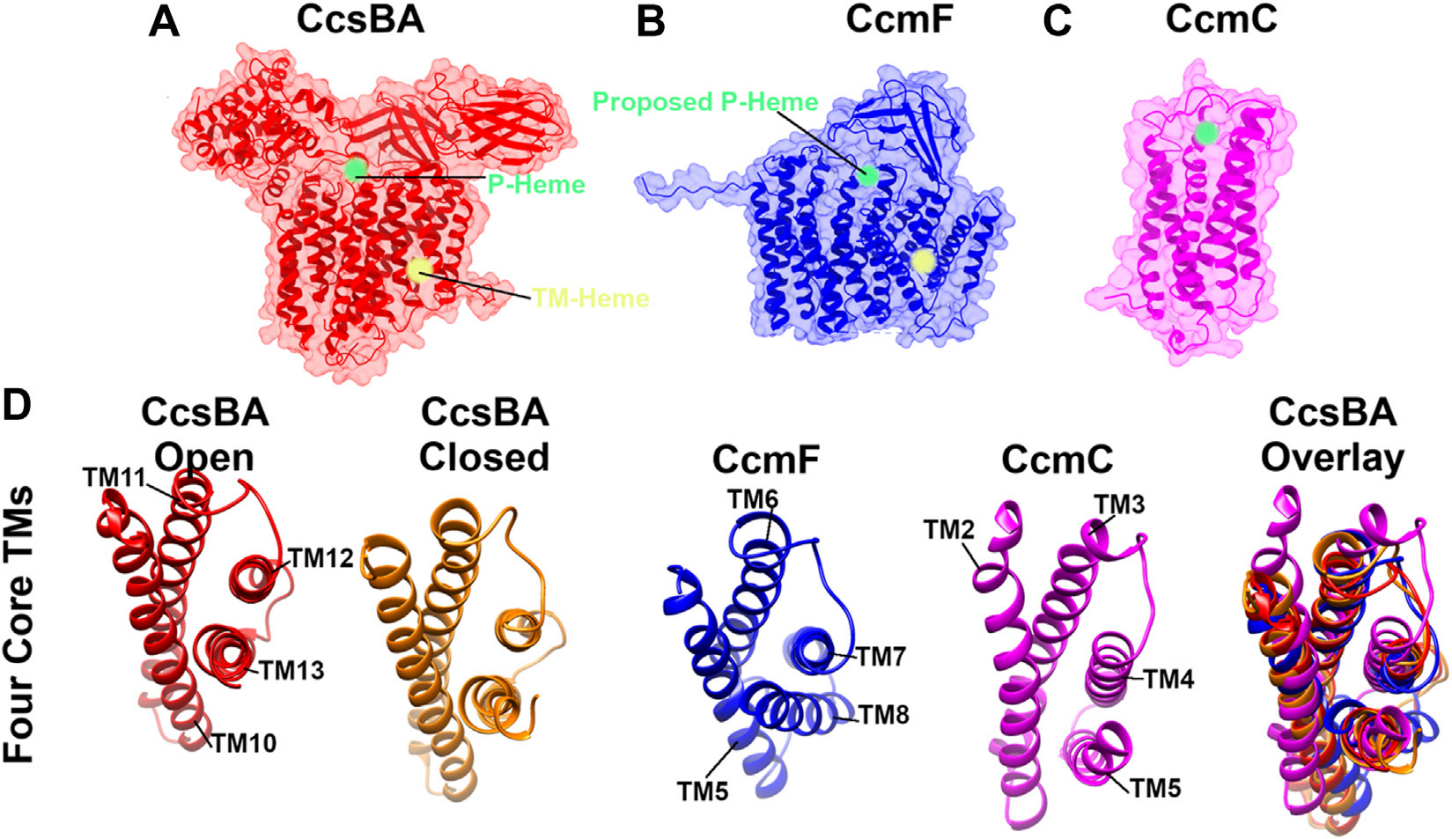
**Overview of structures of WWD Proteins.** The heme (P-heme, *green*) in the periplasmic binding site is formed by the WWD domain as well as two histidine ligands (called P-His1 and P-His2). Heme that lies within the inner leaflet plane (TM-heme, *yellow*) is liganded by two conserved histidines in TMs (called TM-His1 and TM-His2); CcmC is lacking these TM histidines. *A*, structure of CcsBA (open conformation) shown in both *cartoon* and van der Waals surface (25). *B*, structure of CcmF shown in *cartoon* and van der Waals surface (24). *C*, structure of CcmC shown in *cartoon* and van der Waals surface (26). *D*, four core TMs (transmembrane helices) are similar among WWD proteins. TM, transmembrane.

System I is comprised of eight Ccm proteins (in *Escherichia coli*), and works in two general steps (Fig. 1*A*): the first is to transport heme *via* CcmABCD, an ABC transporter complex, and attach the heme to CcmE, then release holoCcmE. Recent cryo-EM structures of CcmABCD showed where exogenously added heme bound to the CcmC WWD/P-His domain (26). The various structures ellucidated how ATP hydrolysis by CcmA would release the holoCcmE from CcmC into the periplasm to chaperone its heme to the CcmF/H cyt c synthase. CcmABCD structural studies of the ATP-dependent release of holoCcmE from CcmABCD may be the best understood release mechanism for the family. In the cases of CcsBA and CcmF/H (the cyt c synthases), mechanisms of release of holoCXXCH remain speculative. Still unknown for CcmCD is how heme moves to the periplasmic WWD/P-His domain (Fig. 2, *C* and *D*), whether *via* a channel through the CcmC protein or from the outer leaflet. Here, it is important to note that heme transport only requires CcmCD, not the entire CcmABCD complex (20, 27, 28). It has been proposed that after CcmE binds to CcmCD, covalent CcmE-heme adduct formation (CcmE His130 to the 2-vinyl of heme) favors oxidized heme (Fe^+3^) (1, 29). CcmE has been termed a periplasmic heme chaperone (30), and its adduct formation of heme to His130 is unique in biology (30, 31, 32, 33, 34, 35). How the heme is oxidized before adduct formation is also an open question. Once released by the CcmABCD complex, holoCcmE chaperones the Fe^+3^ heme to the active site of CcmF/H for step 2, cyt c synthesis (Fig. 1*A*) (36).

The structures of both CcsBA (25) and CcmF (24) provide a deeper understanding of heme trafficking and cyt c synthase mechanisms. For CcmF, we examine the published crystal structure in the context of our CcsBA cryoEM structures, the open state of which has endogenous heme at the active site, its WWD/P-His domain (Fig. 1*B*). This analyses and other evidence are collectively used to describe mechanisms of trafficking holoCcmE (heme) to the CcmF/H cyt c synthase active site (the WWD/P-His domain). Our analyses here indicates that heme (*via* holoCcmE) enters CcmF from above, *via* the soluble periplasmic milieu, directly into the WWD/P-His domain. We call this the periplasmic entry model, with heme delivery by holoCcmE directly to the active site. In contrast to the heme transport function of CcsBA (Fig. 1*B*), we have proposed a reduction function for CcmF whereby the heme in holoCcmE is reduced (to Fe^+2^) prior to attachment to CXXCH(29). CcmF thus is a cytochrome itself, using its stable transmembrane (TM)-bound heme to reduce the incoming heme of holoCcmE. We discuss key residues involved in the multifunctional features of CcmF/H, which can be considered a heme oxidoreductase and cyt c synthase machine.

CcsBA has been referred to as a bifunctional heme transporter and cyt c synthase machine (37, 38). CcsBA transports reduced heme (Fe^+2^) from inside to outside, and attaches it to CXXCH (Fig. 1*B*). We explore CcsBA from a wide distribution of organisms (Fig. 1*C*), fitting key conserved regions into cryo-EM densities to scrutinize the structural basis for universal mechanisms of these functions. This includes periplasmic chamber domains for access to the CXXCH motif, the binding of CXXCH, and conserved heme binding and export features. We structurally define two pockets for the two thiols of the CXXCH substrate, which also facilitate histidine (of CXXCH) liganding to the substrate heme at the active site. Above the active site we observe a beta stranded domain in all CcsB/A proteins. We suggest this “beta cap” functions to occlude the active site when the substrate heme is not present.

## Results

### CcsBA and conserved structural features of the subfamily: A heme transporter and cyt c synthase machine

We begin with CcsBA because its structures, with endogenous heme bound at the active site, offers insights that are later applied to CcmF/H. The protein topology and primary sequence of CcsBA from *Helicobacter hepaticus* is displayed in Fig. S1*A*, with conserved residues in the subfamily colored red. The topology is used as a framework for discussing three dimensional elements critical for function, some of which have never been addressed.

As background, early genetic studies defined *ccsB* and *ccsA* involvement in cyt c synthesis in model organisms: *ccsA* and *ccsB* (ccs1) in *Chlamydomonas reinhardtii* chloroplasts (39, 40, 41, 42, 43), *ccsA* (*resA*) and *ccsB* (*resB*) for *Bacillus subtilis* (44, 45), *ccsA* and *ccsB* from *Bordetella pertussis* (46, 47, 48). The *ccsBA* gene from a small cadre of subfamily members, such as *H. hepaticus*, have a natural fusion of the *ccsB* and *ccsA* genes, forming a large fused membrane protein (hence called CcsBA). This natural fusion has facilitated overexpression, GST-tagging and hexahistidine tagging, and purification of CcsBA in recombinant *E. coli* (37, 49). The fused recombinants are active in *E. coli*, able to attach heme to a wide variety of cloned, periplasmic c-type cytochromes (50). These recombinant studies in the last 14 years have led to biochemical and now structural analyses, including *in vitro* reconstitution of the heme attachment process by purified CcsBA (51). We focus here on the structural lessons of the active *H. hepaticus* CcsBA protein, as applied to a diverse set of CcsBA (fused) and CcsB-CcsA complexes (unfused). When referring to both, we use CcsB/A. Fig. S1 describes further the fused members of CcsBA (*Helicobacter*, Wolinella, *Bacteroides* in this study).

From cryo-EM studies, one of the two CcsBA structures has heme only in the TM-heme site (Fig. 1*B*). This conformation has a closed periplasmic chamber above the active site, thus is called the “closed” conformation or state. The other structure has heme present in both the TM-heme site and in the external WWD/P-His site. The periplasmic chamber in this conformation is “open” with access to CXXCH, which will bind adjacent to the heme in the WWD/P-His site.

Major questions on CcsBA concern what features are completely conserved in the subfamily, from Gram negative epsilon proteobacterium (*Helicobacter*) to beta proteobacterium (*B. pertussis*) to Gram positive (*Mycobacterium tuberculosis*, *B. subtilis*) to cyanobacteria and chloroplasts. We address whether all elements are present in both the fused CcsBA and two-subunit unfused CcsB-CcsA complexes. As a convenient flow of the structural analyses, we begin with internal (cytoplasmic) heme as it is transported through CcsBA to the external active site, where heme is attached to CXXCH and the holocyt c released for proper folding.

#### A cytoplasmic heme vestibule for entry from ferrochelatase

The general architecture of CcsBA proteins appears to have particularly conserved features, although some of the CcsB/A proteins have a different number of predicted TMs (Table S1). RoseTTAFold-derived structures for CcsB/A proteins showed excellent overall fits into the *H. hepaticus* cryo-EM open conformation, with individual differences in TMs in Table S1 acting as outliers to the densities. It is clear that the core set of 4 TMs that encompass the TM heme site, channel, and WWD/P-His heme site are conserved, as they are also present in CcmC and CcmF (Fig. 2*B*). In *H. hepaticus* CcsBA these are TM10-13.

A key question for any transporter is the initial entry point of the substrate. For CcsB/A, heme enters from the cytoplasmic side (or inner leaflet), moving to the TM heme site that is defined by two TM histidine ligands (called TM-His 1 and 2). Because no CcsBA conformation was uncovered that has heme only in the P-heme site, we have proposed that heme in the TM heme site moves through a channel to the P-heme site by a mechanism analogous to the well-known potassium channel (*e.g.*, (52, 53, 54)). That is, heme entering a cytoplasmic vestibule repels the TM-heme into the channel, this incoming vestibule heme then binds to the TM-heme site. Other mechanisms are certainly possible, although a vestibule on the cytoplasmic side of CcsBA that would hold heme is present in *H. hepaticus* densities (Fig. S2*A*).

We have analyzed a diverse set of CcsBA and CcsB/CcsA proteins for the presence of the putative vestibule and cytoplasmic domains that could assist delivery of heme. Our analyses of other ccsBA gene(s) involved the use of RoseTTAFold to predict structures, then to fit them into our cryo-EM densities and analyze. Of the eight CcsBA or CcsB-CcsA proteins chosen (Fig. 1*C*, red highlighted), fused CcsBA proteins possess a vestibule or possible entry site but no other distinguishing domains on the cytoplasmic side. Interestingly, the unfused CcsA-CcsB complexes possess a large cytoplasmic domain that is immediately adjacent to the proposed heme entry site (to the TM-heme site) (Fig. S2*A*). The general architecture of this cytoplasmic domain is similar among the unfused CcsB-CcsA complexes (Fig. S2*B*). The cytoplasmic domain is in large part composed of an additional C-terminal extension of the *ccsB* sequences. We suggest that this additional domain could facilitate heme delivery to the TM-heme site. It is entirely feasible that heme could be inserted directly into the vestibule or directly into the TM-heme site from the ferrochelatase, the final enzyme in heme biosynthesis that inserts the reduced iron. Such an interaction could be heme-driven or by CcsB/A:ferrochelatase protein:protein contacts. Indeed, using the web-based STRING database on protein:protein interactions, we discovered that in *H. hepaticus*, ferrochelatase is predicted to interact with CcsBA (Fig. S2*C*).

#### Two heme binding sites, beginning with the TM-His (TM heme) site

The first heme liganded binding site in CcsBA has been demonstrated to require two histidines that are present in TM12 (TM-His1) and TM3 (TM-His2) (Fig. S1). Mutation of these two histidines results in low heme levels in purified CcsBA variants, and disrupts function. Moreover, His to Gly or Ala changes are corrected for *in vivo* function when these recombinant CcsBA variants in *E. coli* are grown in the presence of exogenous imidazole. This chemical complementation suggests two stable ligand sites that can bind the free imidazole and thus correct heme binding. Importantly, less than 10% of the heme remains with the TM-His variants upon purification. This suggests that a vestibule would have a weak affinity for heme and that heme must go through the TM-site to the WWD/P-His site. All CcsBA proteins possess TM-His1 and TM-His2, with TM-His2 present in CcsB and TMHis1 in CcsA.

The eight CcsBA predicted (RoseTTAFold) structures fit the densities surrounding the TM-heme binding site, including the TM-His1 and TM-His2 residues (Fig. S3). In each case the two TM histidines are easily positioned as axial ligands to the heme, results that suggest a highly conserved heme transport process, with TM-heme localized in the same plane as the inner leaflet of the bilayer.

#### A “channel” for heme

Biochemical results noted above, as well as analysis of the *H. hepaticus* CcsBA cryo-EM structures, indicate that heme moves from the TM-heme site to the external WWD/P-His site. Consistent with this conclusion is the presence of a channel in both the open and closed conformations in the cryo-EM densities. Examination of the eight CcsBA proteins fit to these densities all show this channel (Fig. S4), which encompasses the four core TMs (Fig. S4). The view is from the periplasm (top), looking down toward the TM-heme (green). As will be discussed below, this channel in CcmF is occluded by two conserved tryptophan side chains (W214 and W251), that are absent in CcsBA. The importance of these tryptophans is discussed in the section on the CcmF/H machine.

#### The external heme binding site (WWD/P-His) as the active site to attach heme to CXXCH

Various experiments have shown that the WWD/P-His domain constitutes a heme binding domain that faces the periplasmic space. Both histidines are required for *in vivo* and *in vitro* cyt c synthase function. A novel cysteine scanning mutagenesis of the WWD domain residues resulted in covalent crosslinking to endogenously bound heme (22). That is, when a cysteine replaces certain WWD residues, one of the vinyls of heme covalently attaches the heme (presumably *via* thioethers) to the CcsBA protein. These cys/heme crosslinks were detected and mapped in both CcsBA and CcmC, with results suggesting a conserved common structural motif near the heme vinyl groups.

Analyses of the CcsBA open conformation with endogenous heme in the external binding site indeed confirms the above predictions from biochemical results. The WWD/P-His domain binds heme and represents the active site; this heme will be attached to the CXXCH acceptor. Both periplasmic histidines act as ligands to the heme iron in this state. Thus, the structure of CcsBA (open state) is the only family member so far with a resolvable P-His2 loop and a P-His2 ligand, no doubt stabilized by the heme liganding. We have proposed that the P-His2 loop is flexible, able to move in and out of the active site (25). This is a key element of the heme binding, attachment, and release mechanisms of all three family members, consistent with an unresolvable P-His2 loop in the CcsBA closed state (*i.e.*, with no P-heme).

We used the *H. hepaticus* open conformation density for fitting the other CcsB/A proteins in this active site (Fig. S5, *A* and *B*). It is clear that P-His1 of each CcsB/A fit into the densities (purple in Fig. S5*A*). Although the P-His2 imidazole can be envisioned as a ligand (orange in Fig. S5*A*), the P-His2 loop itself is less poorly predicted by RoseTTAFold, supporting the suggestion of a flexible loop. The P-His1 residue is at the outer leaflet of TM 10 helix, with the P-His1 loop between TM9 and 10. The overall structures in this active site, including core TMs are strikingly similar in all CcsB/A proteins.

Fig. S5*B* shows the same site after computationally removing the P-His2 loop so that the WWD domain can be visualized (green). In fact, the cryo-EM structures show the WWD interaction with heme is nearly identical in CcsBA (open) and the CcmC with added heme. Even more remarkable is that the aforementioned residues in the WWD that cross-linked heme (after cysteine substitution) are adjacent to the 2 or 4 vinyl groups of heme (21, 26). Fitting of each CcsB/A into the open state density (Fig. S5*B*, green density) illustrates the conservation of the WWD domain. We have argued previously that the WWD domain is for binding heme and likely for releasing it once the covalent thioether bonds are formed. Additionally, the WWD side chains may be conserved to aid the chemistry of covalent bond formation (thioethers for CXXCH substrate and the CcmE His130 adduct for CcmC). Even though these are spontaneous reactions, the WWD environment may optimize them.

#### A proposed conserved binding site for CXXCH at the active site

**Figure 3:**
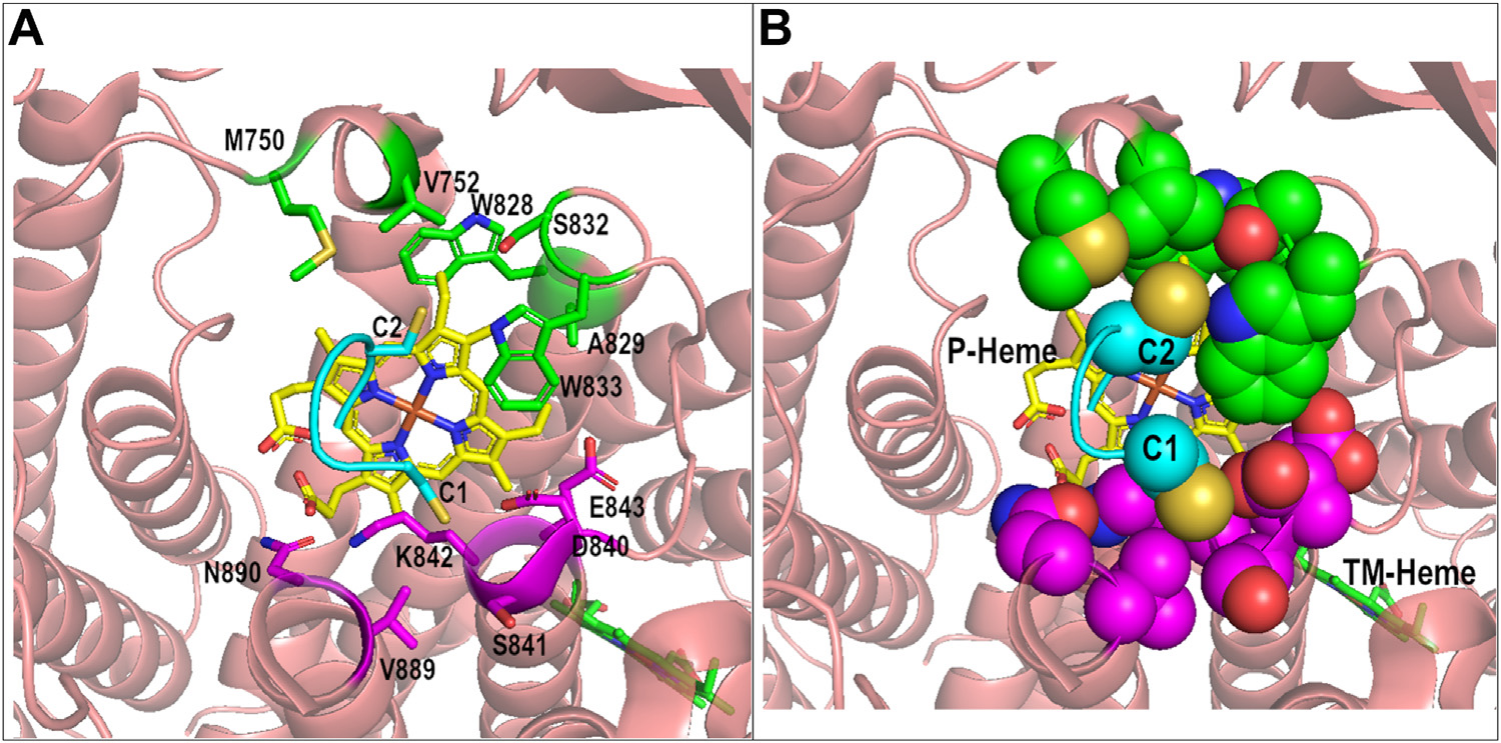
**Docking of CXXCH into CcsBA active site (human cyt c CSQCH, cyano).** C1 represents the first cys of CXXCH and C2 the second. As described in Experimental procedures, the program Autodock Vina was used. *A*, stick, (*B*) space-filling.

We used our CcsBA open state density and the program Autodock Vina to search for a possible CXXCH binding site, with the histidine of CXXCH replacing P-His2 as axial ligand. Here, we note highly conserved residues in the putative CXXC binding site. Figure 3, *A* and *B* displays the human cyt c sequence CSQCH docked into the active site above the external heme in the CcsBA open state. Two pockets are present in which the first cysteine (C1) and second cysteine (C2) are docked. These are adjacent to vinyl 2 and 4, respectively, thus a mechanism for the stereochemical attachment is revealed by this binding and the histidine liganding (of his in CXXCH). For reference in the topological sequence of CcsBA and numbering, refer to Fig. S1 topology for conserved residues that form each pocket of the CXXCH binding site. The C1 pocket (Fig. 3*A*) is formed by residues (colored purple) that include the outer TM13 (conserved V889 and N890) as well as residues at the C terminus of the WWD sequence (conserved D840, S841, K842, and E843). The C2 pocket (Fig. 3*A*) is formed by residues (colored green) that includes a periplasmic loop between TM9 and 10 (conserved LMPVL, 749–753) as well as conserved residues at the N terminus of the WWD sequence (conserved W828, A829, S832, and W833). Conservation of nearly all these residues suggests a highly conserved binding site for the CXXCH substrate (Figs. 3 and S1).

In addition to the canonical cyt c heme binding motif CXXCH, some bacteria possess very rare c-type cytochromes that have alternative substrates, CXXCK and CX_15_CH. In these unusual cases, dedicated CcsBA (or CcmF/H) machines are present, in addition to the canonical CcsBAs that recognize CXXCH. *Wolinella succinogenes* is one example of these bacteria, and its three substrates are recognized by three different fused CcsBA proteins (55, 56). They have been included in the present review, and as shown above they possess all important features conserved in CcsB/As. It appears that they also possess the two pockets that we propose recognize the cysteines of the CXXC or CX_15_C motifs. For the CX15C substrate, some other aspect of this long X15 “loop” must be recognized and required. For the CXXCK, it may be that its cognate CcsBA has a weakly bound P-His2 imidazole that can be replaced by the lysine of CXXCK but not by the canonical histidine (of CXXCH). Structures of these unusual CcsBA proteins could address such questions on alternative substrate recognition.

#### A regulated chamber in fused CcsBA proteins, and a conserved beta cap domain in all CcsB/A proteins

**Figure 4:**
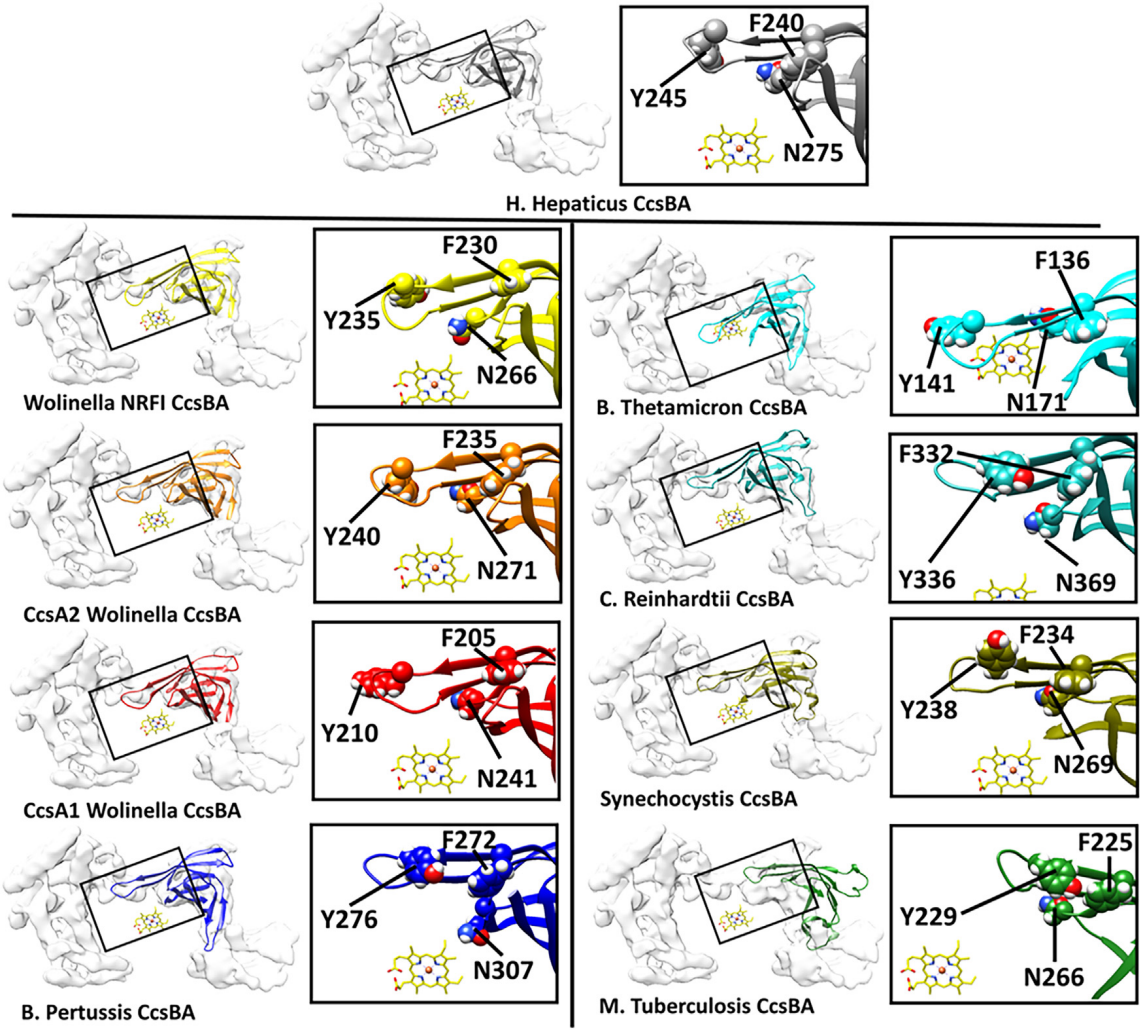
**The periplasmic beta cap domain of CcsB/A proteins.** The region that closes over the active site P-heme (when no heme is present) is marked with a *rectangle*, and three conserved residues are displayed in space-filling format.

The CcsBA open and closed conformations from *H*. *hepaticus* revealed a large periplasmic chamber which opens for CXXCH substrate access only when heme is at the active site. We discussed the molecular basis for the opening upon heme binding to the WWD/P-His site and speculated that closing could protect the channel and/or TM-heme (25). Since the structures are from the fused CcsBA of *H. hepaticus*, we here analyze whether the other fused CcsBA proteins and the unfused CcsB-CcsA complexes might also have a periplasmic chamber (Fig. S6*A*). As in *H. hepaticus*, fused CcsBAs have chambers (Fig. S6*A*, Wolinella, *Bacteroides thetaiotaomicron*). Part of this chamber is comprised of what we here designate the beta cap domain, a more conserved region that appears to occlude (cap) the active site upon closing (see ovals in Fig. S6*A*). The unfused CcsB-CcsA complexes possess a smaller periplasmic region, all possessing the CcsB beta cap (Fig. 4).

We propose that the periplasmic beta cap, consisting largely of beta sheets with a few conserved residues, occludes the active site when heme is not present. It is notable that the fused CcsBA proteins have two periplasmic domains (PD1 and PD2) that are separated by two TMs (Fig. S1). PD1 and PD2 are conserved in architecture for each of the fused CcsBA proteins. However, the unfused CcsB-CcsA complexes possess various lengths of periplasmic sequences, some have PD1 with largely the beta cap in CcsB (Fig. S6*A*), the beta cap representing the conserved domain structurally and likely functionally. Figure 4 focuses on the beta cap of each CcsB/A protein. Of particular note is a conserved loop of the beta cap that sits directly above the active site in the closed state, moving a few angstroms off in the open state. Fig. S6*B* shows the best-fit analyses of the beta cap sequences, with the loop region labeled. Within the loop is a conserved tyrosine (Tyr245 in *H. hepaticus*) and phenylalanine (Phe 240 in *H. hepaticus*) that begins the loop (Fig. 4). These may act as true plugs to the channel, or they may control access to the active site only when heme is present. Although occlusion of the active site is one possible function for the beta cap (and chambers) it might also play a more active role in the mechanisms of substrate access and/or release of the holocyt c product. The conserved residues in this domain would be the logical starting point to investigate activity requirements, CXXCH access, holocyt c release. Recent *in vitro* reconstitutions of CcsBA will be useful in unraveling functions of the beta cap and chamber, guided by the structures (51).

### CcmF/H subfamily and conserved structural features of the subfamily: A heme oxidoreductase and cyt c synthase machine

The CcmF/H complex represents the system I cyt c synthase, although two key differences in mechanism to CcsBA have been proposed: (1) the donor heme for the synthase reaction is not transported from the TM heme site but instead comes from periplasmic holoCcmE, (2) a function for a “stable” (accessory) heme in the TM site is to reduce the external “donor” heme at the CcmF/H active site (from Fe^+3^ to Fe^+2^). We previously have suggested the chemistry behind formation of the His130 adduct to heme vinyl 2 in CcmE, favoring oxidized heme (1, 20, 29). Since oxidized holoCcmE (Fe^+3^) is the substrate for CcmF/H, we proposed that the role for the stable TM-heme in CcmF is to reduce holoCcmE at the active site (1, 20, 29). Such an oxidoreductase activity would eject the His130 adduct (of CcmE) from the donor heme, as well as leaving Fe^+2^ heme for the cyt c synthase reaction. A CcmF crystal structure begins to elucidate and confirm some of these biochemical results. We focus on two major points of the CcmF structural study, how holoCcmE trafficks to the CcmF/H active site, and an electron transport conduit within the CcmF/H machine.

#### Models for heme (in holoCcmE) trafficking to the external active site (WWD/Phis) of CcmF: A periplasmic entry model or leaflet buoy model

**Figure 5:**
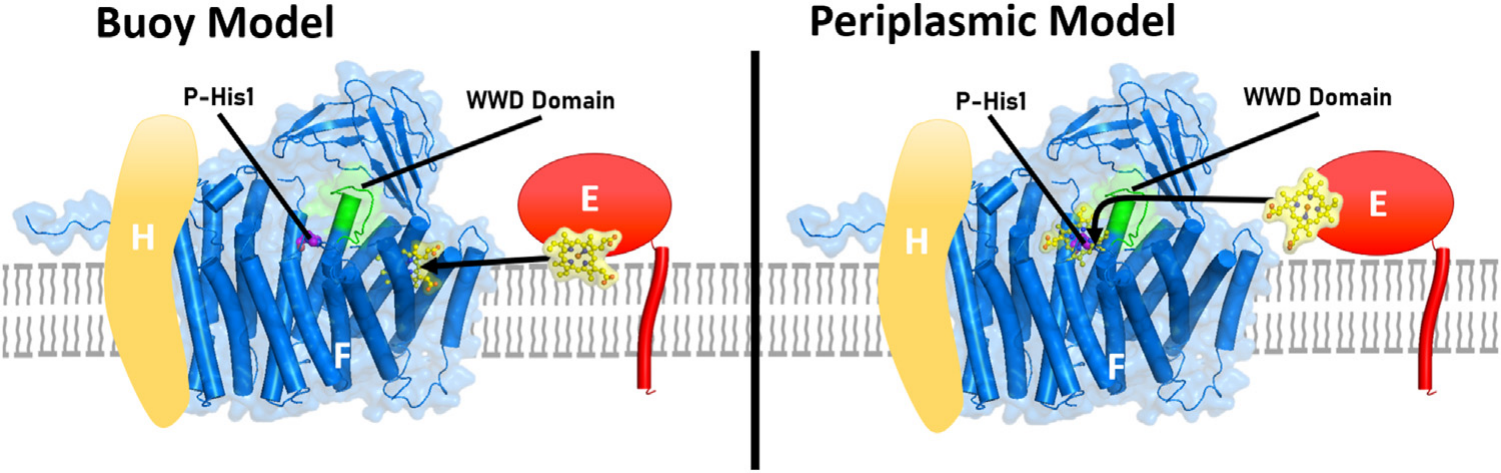
**Mechanisms of trafficking of holoCcmE into the CcmF active site.** In the Buoy leaflet model, as proposed by Brausemann *et al.* (24), heme in CcmE enters a pore in CcmF *via* the outer leaflet. The periplasmic model is proposed in this study, based on the evidence detailed in the text. Labeled are the P-His 1 ligand and the WWD domain.

**Figure 6:**
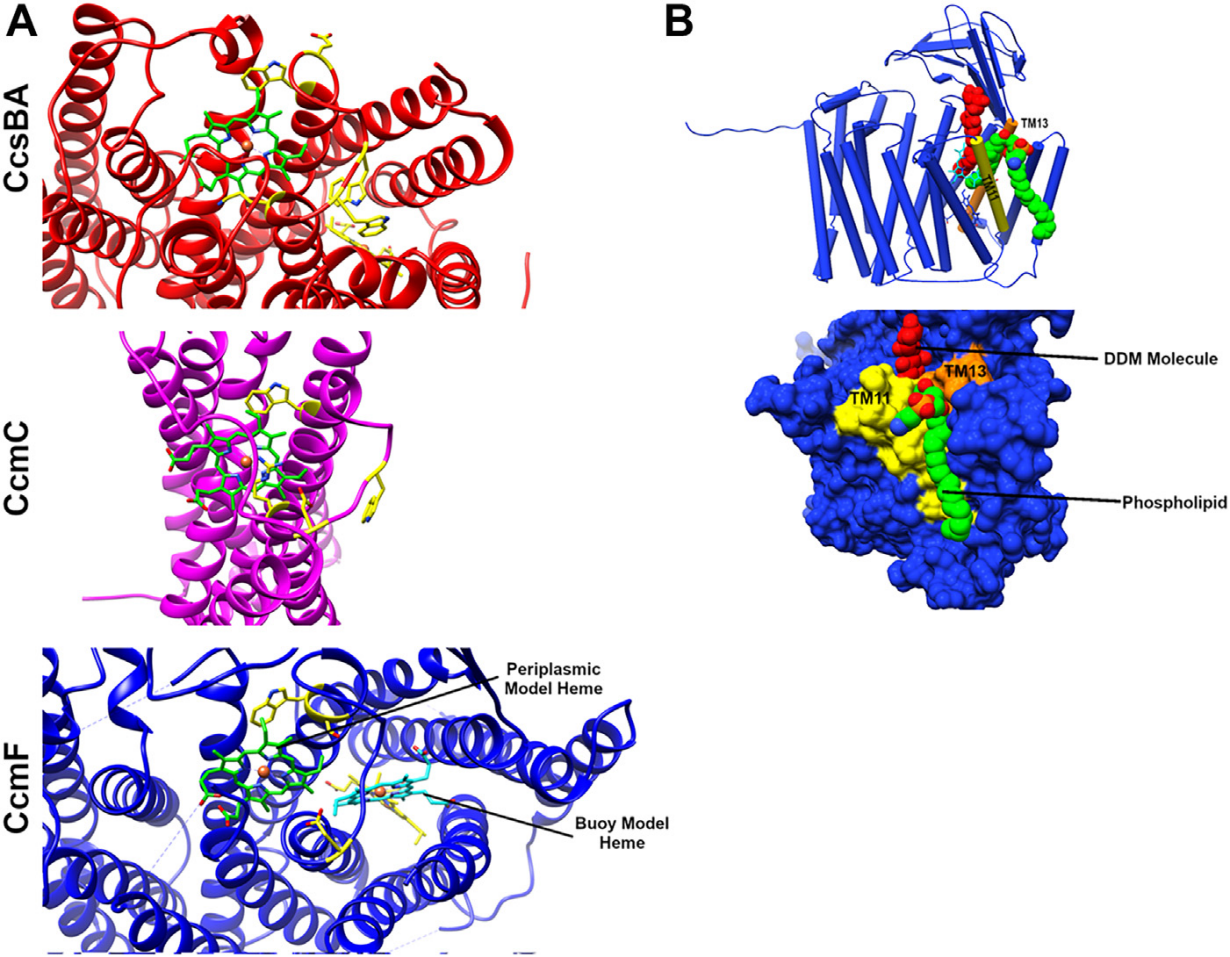
**Locations of P-heme in WWD proteins.***A*, heme (*green*) in the active sites of CcsBA and CcmC as determined by CryoEM studies, and the proposed positions of heme in CcmF by the periplasmic model or buoy model, as discussed in the text. Views are from the *top*, periplasm looking toward the cytoplasm, where the TM-heme in CcsBA and CcmF are partially visible (*yellow*). Amino acid residues shown in *yellow*, *stick* views, have been shown experimentally to crosslink to the P-heme when changed to cysteinyl residues. CcsBA, CcmC, and CcmF crosslinks in these WWD domain studies were as follows: CcsBA: W828, D831, W837, W439, K142. CcmC: W114, W123, D126, R128. CcmF: W227, S228, and D241. *B*, the location of where heme (*magenta*) is proposed in the buoy model also showing the presence of a dodecyl maltoside (DDM) detergent molecule and a phospholipid (from (24)). TM, transmembrane.

The structure of CcmF confirmed that the TM stable heme, called an accessory heme by Brausemann *et al.* (24), is liganded by the two TM histidines (in the inner leaflet on TM7 and TM14.) We have previously termed these TM-His1 and TM-His2. Unfortunately, the CcmF crystals did not possess a heme (from CcmE) in the external WWD/P-His site. This raised the question of how heme in holoCcmE might traffick to the active site formed by WWD/P-His. The authors proposed a leaflet “buoy” model whereby heme attached to CcmE enters from an outer leaflet opening in CcmF (Fig. 5). The outer leaflet opening is between TMs 11, 13, and 14, and it leads to what the authors termed a “vestibule” for heme (Figs. 5 and 6). Brausemann *et al.* (24) computationally docked the heme into this vestibule/cleft, suggesting that entry displaces the residues of the WWD domain (W172 and W240). As highlighted in the News and Views by Brown and Iverson (57), this leaflet “buoy” delivery model is based on the large membrane-facing vestibule (cleft), proposing that heme in CcmE trafficks in the outer leaflet of the membrane through this pore (like a “buoy”). Here, we propose an alternative “periplasmic” delivery model where heme in holoCcmE enters CcmF from above, *via* the periplasm (Fig. 5). In this case, holoCcmE is a soluble chaperone (with the exception of its single TM that tethers it to the cytoplasmic membrane.) We favor the “periplasmic” delivery model of trafficking based on the following four observations:

1. In the CcmF leaflet entry model, heme docked in the vestibule/cleft is located at least 17 Å from P-His1 (Fig. 5). Although the authors suggest a conformational change for P-His 1 to move 17 Å (24), P-His1 in CcsBA (Fig. 6*A*) is in the same location whether closed (no P-heme) or open (P-heme present). Likewise, in CcmC, the P-His1 imidazole position is not appreciably changed in various CcmC states and to do so would require significant movement of the entire TM in which P-His1 is located (Fig. 6*A*). Favoring the periplasmic model, there is access for heme (holoCcmE) from above in CcmF at the homologous site in CcsBA (Fig. 6*A*), without envisioning a 17 Å conformational change, and such that P-His1 liganding would occur without major movements. For the periplasmic entry, the WWD residues also would be located similarly to those in CcsBA and CcmC (Fig. 6*A*). Furthermore, the leaflet buoy entry model proposes that heme moves the WWD and is in fact on the opposite side of the WWD domain shown for CcsBA.
2. The CcmF crystal structure (24) has a dodecyl maltoside (molecular weight 510 Da) detergent molecule completely engulfed in the vestibule/cleft where it appears heme was molecularly docked (Fig. 6*B*). Part of the hydrophoblic tails of a surface phospholipid also enters the putative vestibule (Fig. 6*B*). Although Brausemann *et al.* do not describe the details of heme docking, it seems likely they have computationally removed the dodecyl maltoside and phospholipid before molecular docking. Thus, a question remains on whether there is a vestibule/cleft and opening under physiological conditions (*i.e.*, active). It may be pertinent that theoretical CcmF structures using RosseTTAFold (58), alphafold2 (59) and alphafold2complex (60) are architecturally very similar to the experimental structure, but the pore/leaflet entry site and the vestibule are not large enough for heme (Fig. S7).
3. In the buoy leaflet model, CcmE is proposed to chaperone its heme via the outer leaflet, while the heme is submerged in the membrane like a buoy (24). We and others have demonstrated that when the single TM is deleted from CcmE (called CcmE∗), it still has heme attached and is released by the CcmABCD complex (27, 35). However, the holoCcmE∗ protein is present in the periplasm, not associated with the membrane. The periplasmic shock fraction contains a soluble holoCcmE∗. One would predict that by the leaflet model the heme would tether (buoy) holoCcmE∗ to the membrane, analogous to lipidated proteins.
4. The periplasmic entry model hypothesizes that the polar part of heme (*i.e.*, propionate half) in CcmE enters first into the CcmF active site from above. The vinyl half of heme (adduct to His130) is consequently located near the surface within the CcmF active site formed by WWD/P-His. This makes sense since the vinyls will be attached to the incoming CXXCH, as occurs in CcsBA (from above). This in fact is how the active site heme is oriented in CcsBA (25). Thus, no rotations or flipping of the heme are required for the periplasmic entry model (Fig. 6*A*). Only the P-His2 loop would be needed to move into its position, thus replacing the Tyr134 of the holoCcmE. Since the P-His2 loop is not resolved in the CcmF structure, it is likely flexible. As further support for this orientation and location in the periplasmic model, the Sutherland lab has recently published WWD cysteine/heme crosslinking studies on CcmF (61), where they show similar crosslinking to those observed in CcsBA and CcmC (Fig. 6*A*).

Taken together, we suggest that the “periplasmic entry” model for CcmE, from the periplasmic surface, is consistent with the available data. Based on Cryo-EM structures of CcsBA (25) and structural predictions on CcmF (24) it is likely that CcmF/H cyt c synthase complex will undergo some conformational changes during its activities. However, it remains to be determined at what stages in the process this will occur and whether such large movements proposed by Brausemann *et al.* (17 Å) will take place. Structures of various conformations as the CcmF/H synthase complex, with and without the holoCcmE heme donor, will be needed.

#### An electron reduction conduit to initiate a complex attachment of heme (from holoCcmE) to CXXCH

**Figure 7:**
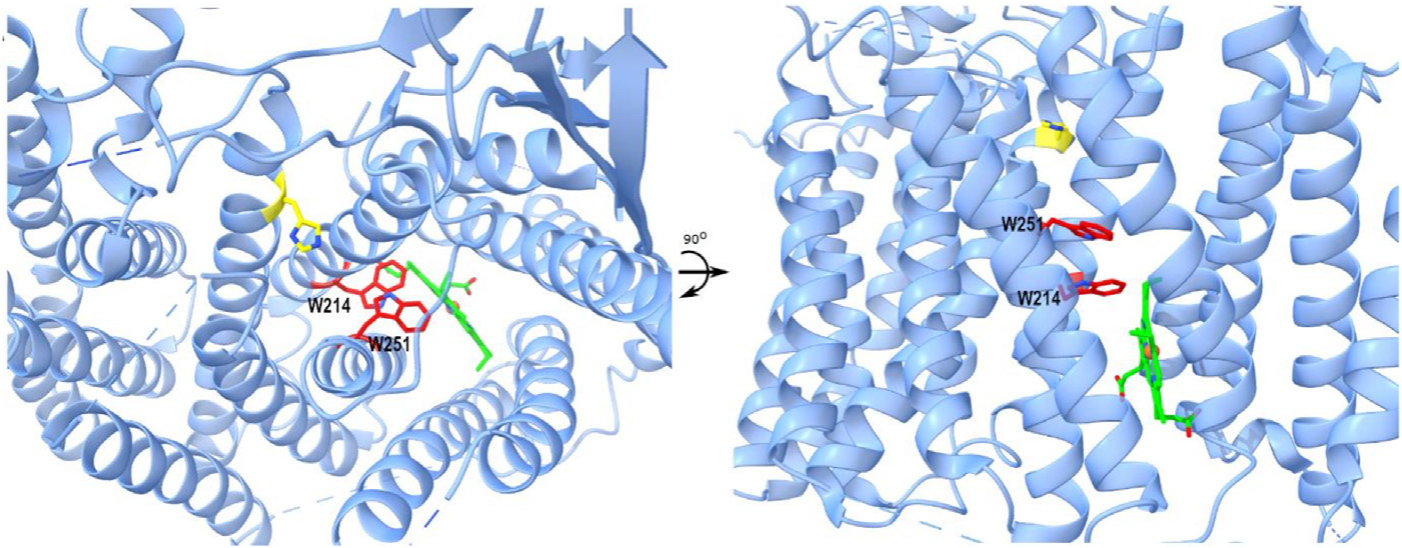
**A heme oxidoreductase conduit in CcmF.***Thermus thermophilus* CcmF crystal structure (from (24)), *top* and *side* views. Shown are two conserved tryptophans (W214 and W251) in the channel between b-heme (*green*) and P-His1 (*yellow*) that represents the general location of the active site (P-heme site where holoCcmE heme enters). In using the P-heme location as proposed in the “periplasmic model” of the CcmF active site (see Fig. 6*A*), the following distances were measured for putative electron transfer: TM-heme to W214: 4.2 Å; W214 to W251: 3.7 Å; W251 to P-heme: 8.4 Å. The edge of heme was used for measurements, not the iron. Using the iron of each heme, distances of TM-heme to W214 is 7.3 Å and W251 to P-heme is 10.3 Å. For the CcmF from *Escherichia coli*, W114 is W216 and W251 is W253. TM, transmembrane.

CcmF/H is a complicated machine whose various activities at the active site are proposed to begin with reduction of the heme in holoCcmE (1). This reduction is initiated by the TM heme, its reduction potential being −147 ± 2 mV at pH 7 (62). The reductant of TM-heme is unknown, possibly a quinone or NADH. Brausemann *et al.* (24) have suggested the involvement of two conserved TM tryptophans in this reduction relay (W214 and W251 in *Thermus thermophilus* CcmF, W216, and W253 in *E. coli* sequence). These two tryptophans not only occlude the channel from TM-heme to P-heme, they are absent in CcsBA. Thus, the proposed order of reduction is as follows: unknown reductant→TM-heme→W214→W251→P-heme. Figure 7 shows a side view and top view of the TM tryptophans, as well as the distances involved between reactants in the conduit. Tryptophans have previously been proposed as intermediates in certain cytochromes (between hemes) and the distances in CcmF are within the accepted electron transfers shown in other systems (*e.g.*, (63)).

The CcmH protein is known to act as a thiol reduction protein (as is CcmG or in its place DTT) for reduction of the cysteines in CXXCH (64, 65, 66, 67). CcmH also likely aids in CXXCH acceptor binding since DTT cannot substitute. A theoretical structure of CcmF/H using alphafold 2 complex places the CcmH thioredoxin site (CXXC) near the WWD/P-His (60). However, an experimental structure of CcmF/H with and without holoCcmE is needed to further address roles of CcmH and active site features. Once the heme from holoCcmE is reduced, and assuming the active site is now bound to a CXXCH acceptor, four reactions are likely to ensue in an unknown order: (1) the histidine of CXXCH replaces the P-His2 (which itself has replaced the Tyr134 of CcmE); (2) it is mandatory that the His130 adduct to the 2 vinyl must be ejected first; (3) the Cys1 (of CXXCH) forms a thioether to the 2 vinyl; (4) a second thioether between Cys 2 of CXXCH forms to the vinyl 4 of heme. We present dynamic views of these activities catalyzed by the CcmF/H machine, from reduction reactions to cyt c synthesis, in Video S1.

Subsequent to formation of both thioethers, the holoCXXCH product (cyt c) is released from the active site. The release mechanism is expected to be similar to CcsBA. We have shown that in human HCCS, the heme distortion produced by two thioether attachments induces release, likely weakening the binding of heme to the cyt c synthase (8). Both CcsBA and CcmF/H likely are aided by this distortion in release. Additionally, we have suggested that one role of the WWD domain is to optimize this release. More studies are needed to verify this contention and understand how and which residues in the domain play a role.

## Discussion

There are three structural features present in all three family members (CcsBA, CcmC, and CcmF) that stand out: a nearly identical core of four TMs, the WWD domain, and two periplasmic histidines (P-His1 and P-His2). Figure 2*A* shows the overall structures of each member, with hemes in each class. Four core TMs form a central channel; in the case of CcsBA and possibly CcmC, this channel allows heme movement from inside to the outside WWD/P-His site. In the case of CcmF, two tryptophans and other side chains occlude the channel. The four core TMs also link the WWD domain, a conserved periplasmic loop that defines the family. P-His1 is localized to the outer TM of one of these four core TMs in each member, thus defining a structural and functional (heme liganding) state for all members. P-His2 is flexible, mobile, and part of a periplasmic loop following the last core TM in all three family members.

In CcsBA, a conserved periplasmic “beta cap” is part of a large chamber in the fused CcsBA proteins, opening only when heme is in the active site (WWD/P-His). Importantly, the beta cap domain is situated directly above the active site (P-heme) in this chamber. For unfused CcsB-CcsA complexes, the beta cap domain is often not part of a chamber, but could also occlude the active site when heme is not present. Interestingly, as pointed out by Brausemann *et al.* (24), CcmF appears to have a periplasmic beta stranded structure (Fig. S8). CcmC does not possess a significant periplasmic domain (19, 26), while both CcsBA and CcmF/H may use their domains to occlude access to CXXCH, each beta cap located directly above the heme. Recent reconstitution of cyt c synthase activities with purified CcsBA (51) will resolve whether these Beta stranded domains are required for capping, substrate binding, and/or release.

Based on our CcsBA structures (25) as well as other lines of evidence described here, we suggest that holoCcmE enters the CcmF active site from above (called the “periplasmic model” of entry). More studies are needed to determine the orders of reactions at the CcmF/H active site once heme is bound, as described above. The mechanisms and path for trafficking of heme in both CcmC and CcmF (*via* CcmE) will require more structures and other experiments.

Release of the heme-attached substrates remains speculative, although the cryo-EM structures of CcmABCD certainly point the way for ATP hydrolysis-mediated movement of the P-His2 loop for release of holoCcmE from CcmABCD (26). In contrast, CcsBA and CcmF/H must have a more subtle basis for release of cyt c (holoCXXCH). They likely use a combination of (i) heme distortion due to thioether formation, (ii) WWD residues to assist (*e.g.*, repulsion of the thioethers), and (iii) ligand switching (the CXXCH histidine replacing the P-His2 ligand). *In vitro* reconstitution (cyt c synthase and release) was recently achieved with purified CcsBA, opening up the possibility that the recognition (of CXXCH) and release features can be dissected.

Interaction of CcmC and CcsBA with heme biosynthetic enzymes, specifically ferrochelatase, may provide direct entry of newly synthesized heme. In general, how heme enters heme transporters and the mechanisms of transport remain open questions. *In vivo* and *in vitro* studies on protein:protein interactions are needed. The studies on mitochondrial protein interactions, including those specifically with ferrochelatase, are a good example (68, 69).

Theoretical structures of the CcmABCD, CcmFH/E, and CcsBA complexes have been predicted using increasingly improved structural prediction programs over the last 5 years (*e.g.*, Gremlin/Rosetta, trRosetta, RoseTTAFold, and alpha2complex) (*e.g.*, (21, 22, 60)). One conclusion we can make in comparing these output to the recent experimental structures is that while the structures are very impressive and closely align, determinations of the detailed binding sites for substrates (heme, CXXCH) require experimental cryoEM or crystallographic structures, often even with and without substrate (*e.g.*, heme in the CcsBA open state).

Finally, a longer term goal of understanding the cyt c biogenesis systems is to control them. For example, *in vitro* biosynthesis (51) might facilitate the attachment of hemes to new (poly)peptides that could be used themselves as electron conduits or for new synthesis reactions. By understanding the rules of substrate recognitions, including attachments, new substrates that bind the active sites or unique recombinant and engineered synthases could lead to new heme enzymes or conduits. As described above, the recombinant systems have already been used to produce unique enzymes with CXXCH-attached hemes (70, 71), the next frontier might now move further from the canonical unfolded CXXCH motif.

## Experimental procedures

### Structural fitting of CcsB/A proteins into the known cryoEM densities of *H. hepaticus* CcsBA

The generation of cryo-EM densities of purified *H. hepaticus* CcsBA preparations has been previously described (open conformation in electron microscopy databank-24941; closed electron microscopy databank-24942) (25). Experimentally determined structures were accessed through the Protein Data Bank under accession codes as follows: CcsBA Open Conformation: 7S9Y, CcsBA Closed Conformation: 7S9Z, CcmF: 6ZMQ, CcmCABCD: 7F04. Unless otherwise stated as a theoretical structure, these structures and densities represent those used in the manuscript for CcsB/A analyses.

All theoretical structures for CcsB/A proteins (other than *H. hepaticus*) were generated through RoseTTAFold (58) using the webserver: https://robetta.bakerlab.org/. In addition to RoseTTAFold, as described, we used WinCoot (72), AutoDock Vina (73), UCSF Chimera (https://www.cgl.ucsf.edu/chimera/), and Pymol (https://pymol.org/2/). For CcsB/A analyses, we first compared the theoretical structures generated to the known cryoEM structure of *H. hepaticus* by overlaying the theoretical structures with the structure of *H. hepaticus* through the PyMOL “align” feature. From here Chimera’s “fit in map” was used to dock the structures into the *H. hepaticus* open electron density. Structures of fused CcsBA (Wolinella NRFI, Wolinella CcsA1, Wolinella CcsA2, and *B. thetaiotamicron*) were modeled using their already naturally fused CcsBA sequence. See Table S1 for information on the *ccsB/A* from each organism. The unfused CcsB/A structures (*B. pertussis*, *Chlamydomonas reinhardtii*, Synechocystis, and *M. tuberculosis*) were modeled with CcsA and CcsB predicted separately. Proteins for these unfused structures were similarly overlayed by PyMOL's “align” feature followed by the Chimera “fit in map” feature placing them into the open map density. The fit to density was further investigated in Coot (https://www2.mrc-lmb.cam.ac.uk/personal/pemsley/coot/) with CcsB in the unfused structures being rigidly docked using WinCoot’s rigid body fit zone to better align CcsB to density and prevent obvious physically improbable overlap with CcsA. All fittings were done rigidly, and only whole peptides were moved to avoid rearrangement of the position of TMs and residues relative to one another. All calculations of surfaces were done in Pymol using the MSMS algorithm. The surfaces were then considered relative to the electron cloud densities of *H. hepaticus* for the figures.

### A binding site in CcsBA cryoEM densities for the CXXCH substrate

As described previously, docking of the CXXCH (human cyt c CSQCH) into the CcsBA structure was performed by removing the P-His2 loop of CcsBA and using AutoDock Vina to dock the CXXCH. The model chosen was the energetically plausible model that best liganded heme (*via* the histidine of CXXCH). In the present study, individual residues in the CXXCH binding site are analyzed and discussed.

### Analysis of the published CcmF crystal structure and theoretical structures

Figures were generated using Chimera, and molecular surfaces were also generated using the surface function within Chimera. Alignment of structures was completed using the “matchmaker function” in Chimera.

The theoretical structure of CcmF was generated using the RoseTTAFold webserver. Heme pore width (pore defined by buoy model) was measured using UCSF Chimera, measuring the largest vertical and horizontal distance of each CcmF pore.

### Video of the CcmF machine

For the video showing the activities of CcmF, the crystal structure of CcmF from Brausemann *et al.* (24) was used. We begin the video with CcmE, only using the heme-attached region of CcmE (H_130_DENY_134_) modeled with WinCoot, and heme positioned according to the periplasmic model of entry, based on CcsBA open state analogies. The flexible loop containing P-His2 was constructed in WinCoot using the sequence of CcmF and modeled based on homology to the flexible loop of the *H. hepaticus* CcsBA structure (open state). After holoCcmE binding, electron transfer/reduction and apoCcmE ejection, CXXCH binding, attachment, and release are modeled, similar to the methods and mechanisms described for the *H. hepaticus* CcsBA structural studies.

## Data availability

All data are contained within the manuscript.

## Supporting information

This article contains supporting information.

## Conflict of interest

The authors declare that they have no conflicts of interest with the contents of this article.

## References

[1] Kranz R.G., Richard-Fogal C., Taylor J.-S., Frawley E.R. (2009). Cytochrome c biogenesis: mechanisms for covalent modifications and trafficking of heme and for heme-iron redox control. Microbiol. Mol. Biol. Rev..

[2] Sanders C., Turkarslan S., Lee D.-W., Daldal F. (2010). Cytochrome c biogenesis: the Ccm system. Trends Microbiol..

[3] Stevens J.M., Mavridou D.A.I., Hamer R., Kritsiligkou P., Goddard A.D., Ferguson S.J. (2011). Cytochrome c biogenesis system I. FEBS J..

[4] Bowman S.E.J., Bren K.L. (2008). The chemistry and biochemistry of heme c: functional bases for covalent attachment. Nat. Product Rep..

[5] Simon J., Hederstedt L. (2011). Composition and function of cytochrome c biogenesis system II. FEBS J..

[6] Kranz R., Lill R., Goldman B., Bonnard G., Merchant S. (1998). Molecular mechanisms of cytochrome c biogenesis: three distinct systems. Mol. Microbiol..

[7] Dumont M.E., Ernst J.F., Hampsey D.M., Sherman F. (1987). Identification and sequence of the gene encoding cytochrome c heme lyase in the yeast Saccharomyces cerevisiae. EMBO J..

[8] Babbitt S.E., San Francisco B., Mendez D.L., Lukat-Rodgers G.S., Rodgers K.R., Bretsnyder E.C. (2014). Mechanisms of mitochondrial holocytochrome *c* synthase and the key roles played by cysteines and histidine of the heme attachment site, Cys- *XX* -Cys-His. J. Biol. Chem..

[9] Babbitt S.E., Hsu J., Kranz R.G. (2016). Molecular basis behind inability of mitochondrial holocytochrome *c* synthase to mature bacterial cytochromes: DEFINING A CRITICAL ROLE FOR CYTOCHROME c α HELIX-1. J. Biol. Chem..

[10] San Francisco B., Bretsnyder E.C., Kranz R.G. (2013). Human mitochondrial holocytochrome c synthase’s heme binding, maturation determinants, and complex formation with cytochrome c. Proc. Natl. Acad. Sci. U. S. A..

[11] Khan A.A., Quigley J.G. (2011). Control of intracellular heme levels: heme transporters and heme oxygenases. Biochim. Biophys. Acta Mol. Cell Res..

[12] Yuan X., Fleming M.D., Hamza I. (2013). Heme transport and erythropoiesis. Curr. Opin. Chem. Biol..

[13] Reddi A.R., Hamza I. (2016). Heme mobilization in animals: a metallolipid’s journey. Acc. Chem. Res..

[14] Hanna D.A., Harvey R.M., Martinez-Guzman O., Yuan X., Chandrasekharan B., Raju G. (2016). Heme dynamics and trafficking factors revealed by genetically encoded fluorescent heme sensors. Proc. Natl. Acad. Sci. U. S. A..

[15] Dai Y., Sweeny E.A., Schlanger S., Ghosh A., Stuehr D.J. (2020). GAPDH delivers heme to soluble guanylyl cyclase. J. Biol. Chem..

[16] Sweeny E.A., Singh A.B., Chakravarti R., Martinez-Guzman O., Saini A., Haque M.M. (2018). Glyceraldehyde-3-phosphate dehydrogenase is a chaperone that allocates labile heme in cells. J. Biol. Chem..

[17] Babbitt S.E., Sutherland M.C., Francisco B.S., Mendez D.L., Kranz R.G. (2015). Mitochondrial cytochrome c biogenesis: no longer an enigma. Trends Biochem. Sci..

[18] Beckman D.L., Trawick D.R., Kranz R.G. (1992). Bacterial cytochromes c biogenesis. Genes Dev..

[19] Goldman B.S., Beck D.L., Monika E.M., Kranz R.G. (1998). Transmembrane heme delivery systems. Proc. Natl. Acad. Sci..

[20] Richard-Fogal C., Kranz R.G. (2010). The CcmC:heme:CcmE complex in heme trafficking and cytochrome c biosynthesis. J. Mol. Biol..

[21] Sutherland M.C., Jarodsky J.M., Ovchinnikov S., Baker D., Kranz R.G. (2018). Structurally mapping endogenous heme in the CcmCDE membrane complex for cytochrome c biogenesis. J. Mol. Biol..

[22] Sutherland M.C., Tran N.L., Tillman D.E., Jarodsky J.M., Yuan J., Kranz R.G. (2018). Structure-function analysis of the bifunctional CcsBA heme exporter and cytochrome *c* synthetase. mBio.

[23] Lee J.-H., Harvat E.M., Stevens J.M., Ferguson S.J., Saier M.H. (2007). Evolutionary origins of members of a superfamily of integral membrane cytochrome c biogenesis proteins. Biochim. Biophys. Acta Biomembr..

[24] Brausemann A., Zhang L., Ilcu L., Einsle O. (2021). Architecture of the membrane-bound cytochrome c heme lyase CcmF. Nat. Chem. Biol..

[25] Mendez D.L., Lowder E.P., Tillman D.E., Sutherland M.C., Collier A.L., Rau M.J. (2022). Cryo-EM of CcsBA reveals the basis for cytochrome c biogenesis and heme transport. Nat. Chem. Biol..

[26] Li J., Zheng W., Gu M., Han L., Luo Y., Yu K. (2022). Structures of the CcmABCD heme release complex at multiple states. Nat. Commun..

[27] Feissner R.E., Richard-Fogal C.L., Frawley E.R., Kranz R.G. (2006). ABC transporter-mediated release of a haem chaperone allows cytochrome c biogenesis. Mol. Microbiol..

[28] Schulz H., Fabianek R.A., Pellicioli E.C., Hennecke H., Thöny-Meyer L. (1999). Heme transfer to the heme chaperone CcmE during cytochrome c maturation requires the CcmC protein, which may function independently of the ABC-transporter CcmAB. Proc. Natl. Acad. Sci. U. S. A..

[29] Richard-Fogal C.L., Frawley E.R., Bonner E.R., Zhu H., San Francisco B., Kranz R.G. (2009). A conserved haem redox and trafficking pathway for cofactor attachment. EMBO J..

[30] Schulz H., Hennecke H., Thöny-Meyer L. (1998). Prototype of a heme chaperone essential for cytochrome c maturation. Science.

[31] Enggist E., Thöny-Meyer L., Güntert P., Pervushin K. (2002). NMR structure of the heme chaperone CcmE reveals a novel functional motif. Structure.

[32] Lee D., Pervushin K., Bischof D., Braun M., Thöny-Meyer L. (2005). Unusual heme-histidine bond in the active site of a chaperone. J. Am. Chem. Soc..

[33] Daltrop O., Stevens J.M., Higham C.W., Ferguson S.J. (2002). The CcmE protein of the c-type cytochrome biogenesis system: unusual *in vitro* heme incorporation into apo-CcmE and transfer from holo-CcmE to apocytochrome. Proc. Natl. Acad. Sci. U. S. A..

[34] Harvat E.M., Stevens J.M., Redfield C., Ferguson S.J. (2005). Functional characterization of the C-terminal domain of the cytochrome *c* maturation protein CcmE. J. Biol. Chem..

[35] Harvat E.M., Redfield C., Stevens J.M., Ferguson S.J. (2009). Probing the heme-binding site of the cytochrome c maturation protein CcmE. Biochemistry.

[36] San Francisco B., Kranz R.G. (2014). Interaction of holoCcmE with CcmF in heme trafficking and cytochrome c biosynthesis. J. Mol. Biol..

[37] Frawley E.R., Kranz R.G. (2009). CcsBA is a cytochrome c synthetase that also functions in heme transport. Proc. Natl. Acad. Sci. U. S. A..

[38] Merchant S.S. (2009). His protects heme as it crosses the membrane. Proc. Natl. Acad. Sci. U. S. A..

[39] Xie Z., Merchant S. (1996). The plastid-encoded ccsA gene is required for heme attachment to chloroplast c-type cytochromes. J. Biol. Chem..

[40] Xie Z., Culler D., Dreyfuss B.W., Kuras R., Wollman F.A., Girard-Bascou J. (1998). Genetic analysis of chloroplast c-type cytochrome assembly in Chlamydomonas reinhardtii: one chloroplast locus and at least four nuclear loci are required for heme attachment. Genetics.

[41] Inoue K., Dreyfuss B.W., Kindle K.L., Stern D.B., Merchant S., Sodeinde O.A. (1997). Ccs1, a nuclear gene required for the post-translational assembly of chloroplast c-type cytochromes. J. Biol. Chem..

[42] Hamel P.P., Dreyfuss B.W., Xie Z., Gabilly S.T., Merchant S. (2003). Essential histidine and tryptophan residues in CcsA, a system II polytopic cytochrome c biogenesis protein. J. Biol. Chem..

[43] Dreyfuss B.W., Hamel P.P., Nakamoto S.S., Merchant S. (2003). Functional analysis of a divergent system II protein, Ccs1, involved in *c*-type cytochrome biogenesis. J. Biol. Chem..

[44] Le Brun N.E., Bengtsson J., Hederstedt L. (2000). Genes required for cytochrome c synthesis in Bacillus subtilis. Mol. Microbiol..

[45] Ahuja U., Kjelgaard P., Schulz B.L., Thöny-Meyer L., Hederstedt L. (2009). Haem-delivery proteins in cytochrome c maturation system II. Mol. Microbiol..

[46] Beckett C.S., Loughman J.A., Karberg K.A., Donato G.M., Goldman W.E., Kranz R.G. (2000). Four genes are required for the system II cytochrome c biogenesis pathway in Bordetella pertussis, a unique bacterial model. Mol. Microbiol..

[47] Feissner R.E., Beckett C.S., Loughman J.A., Kranz R.G. (2005). Mutations in cytochrome assembly and periplasmic redox pathways in Bordetella pertussis. J. Bacteriol..

[48] Kranz R.G., Beckett C.S., Goldman B.S. (2002). Genomic analyses of bacterial respiratory and cytochrome c assembly systems: Bordetella as a model for the system II cytochrome c biogenesis pathway. Res. Microbiol..

[49] Feissner R.E., Richard-Fogal C.L., Frawley E.R., Loughman J.A., Earley K.W., Kranz R.G. (2006). Recombinant cytochromes c biogenesis systems I and II and analysis of haem delivery pathways in Escherichia coli. Mol. Microbiol..

[50] Richard-Fogal C.L., San Francisco B., Frawley E.R., Kranz R.G. (2012). Thiol redox requirements and substrate specificities of recombinant cytochrome c assembly systems II and III. Biochim. Biophys. Acta.

[51] Sutherland M.C., Mendez D.L., Babbitt S.E., Tillman D.E., Melnikov O., Tran N.L. (2021). *In vitro* reconstitution reveals major differences between human and bacterial cytochrome c synthases. Elife.

[52] MacKinnon R. (2003). Potassium channels. FEBS Lett..

[53] Köpfer D.A., Song C., Gruene T., Sheldrick G.M., Zachariae U., de Groot B.L. (2014). Ion permeation in K^+^ channels occurs by direct Coulomb knock-on. Science.

[54] Oakes V., Furini S., Domene C. (2020). Insights into the mechanisms of K(+) permeation in K(+) channels from computer simulations. J. Chem. Theory Comput..

[55] Kern M., Eisel F., Scheithauer J., Kranz R.G., Simon J. (2010). Substrate specificity of three cytochrome c haem lyase isoenzymes from Wolinella succinogenes: unconventional haem c binding motifs are not sufficient for haem c attachment by NrfI and CcsA1. Mol. Microbiol..

[56] Kern M., Scheithauer J., Kranz R.G., Simon J. (2010). Essential histidine pairs indicate conserved haem binding in epsilonproteobacterial cytochrome c haem lyases. Microbiology.

[57] Brown B.L., Iverson T.M. (2021). Handling heme with care. Nat. Chem. Biol..

[58] Humphreys I.R., Pei J., Baek M., Krishnakumar A., Anishchenko I., Ovchinnikov S. (2021). Computed structures of core eukaryotic protein complexes. Science.

[59] Jumper J., Evans R., Pritzel A., Green T., Figurnov M., Ronneberger O. (2021). Highly accurate protein structure prediction with AlphaFold. Nature.

[60] Gao M., Nakajima An D., Parks J.M., Skolnick J. (2022). AF2Complex predicts direct physical interactions in multimeric proteins with deep learning. Nat. Commun..

[61] Grunow A.L., Carroll S.C., Kreiman A.N., Sutherland M.C. (2023). Structure-function analysis of the heme-binding WWD domain in the bacterial holocytochrome c synthase, CcmFH. mBio.

[62] Sutherland M.C., Rankin J.A., Kranz R.G. (2016). Heme trafficking and modifications during system I cytochrome *c* biogenesis: insights from heme redox potentials of Ccm proteins. Biochemistry.

[63] Wang W., Gao Y., Tang Y., Zhou X., Lai Y., Zhou S. (2021). Cryo-EM structure of mycobacterial cytochrome bd reveals two oxygen access channels. Nat. Commun..

[64] Setterdahl A.T., Goldman B.S., Hirasawa M., Jacquot P., Smith A.J., Kranz R.G. (2000). Oxidation-reduction properties of disulfide-containing proteins of the Rhodobacter capsulatus cytochrome c biogenesis system. Biochemistry.

[65] Sanders C., Turkarslan S., Lee D.-W., Onder O., Kranz R.G., Daldal F. (2008). The cytochrome c maturation components CcmF, CcmH, and CcmI form a membrane-integral multisubunit heme ligation complex. J. Biol. Chem..

[66] Turkarslan S., Sanders C., Ekici S., Daldal F. (2008). Compensatory thio-redox interactions between DsbA, CcdA and CcmG unveil the apocytochrome c holdase role of CcmG during cytochrome c maturation. Mol. Microbiol..

[67] Verissimo A.F., Khalfaoui-Hassani B., Hwang J., Steimle S., Selamoglu N., Sanders C. (2017). The thioreduction component CcmG confers efficiency and the heme ligation component CcmH ensures stereo-specificity during cytochrome *c* maturation. J. Biol. Chem..

[68] Obi C.D., Dailey H.A., Jami-Alahmadi Y., Wohlschlegel J.A., Medlock A.E. (2023). Proteomic analysis of ferrochelatase interactome in erythroid and non-erythroid cells. Life (Basel).

[69] Piel R.B., Dailey H.A.J., Medlock A.E. (2019). The mitochondrial heme metabolon: insights into the complex(ity) of heme synthesis and distribution. Mol. Genet. Metab..

[70] Mendez D.L., Babbitt S.E., King J.D., D’Alessandro J., Watson M.B., Blankenship R.E. (2017). Engineered holocytochrome *c* synthases that biosynthesize new cytochromes *c*. Proc. Natl. Acad. Sci. U. S. A..

[71] Mendez D.L., Akey I.V., Akey C.W., Kranz R.G. (2017). Oxidized or reduced cytochrome *c* and axial ligand variants all form the apoptosome *in vitro*. Biochemistry.

[72] Emsley P., Cowtan K. (2004). Coot: model-building tools for molecular graphics. Acta Crystallogr. D Biol. Crystallogr..

[73] Trott O., Olson A.J. (2010). AutoDock Vina: improving the speed and accuracy of docking with a new scoring function, efficient optimization, and multithreading. J. Comput. Chem..

