# Supplementary material for: Structural basis of membrane machines that traffick and attach heme to cytochromes
Source: J Biol Chem. 2023 Oct 10;299(11):105332. doi: 10.1016/j.jbc.2023.105332 (PMC10663686; doi:10.1016/j.jbc.2023.105332)
Supplement: Supplemental Figures S1–S8 and Table S1 [file mmc1.pdf]

Supporting Information

Structural basis of membrane machines that traffick and attach heme to cytochromes.

Jonathan Huynh, Ethan Lowder, Robert G. Kranz*.

Biology Department
Washington University in St. Louis

Contents:

Supp Fig 1. Fused CcsBA machines, where the *ccsB* and *ccsA* genes are fused into a single ORF
Supp Fig 2. Heme access on the cytoplasmic side to enter the TM-heme binding site
SuppFig 3: The TM-heme binding site in CcsB/A proteins is highly conserved.
SuppFig 4: Core channel of CcsBA from the indicated organisms is conserved

SuppFig 5A: P-His ligands in of CcsBA active site are conserved
SuppFig 5B: Structures of the WWD domain at the CcsBA active site are conserved

SuppFig 6A: Periplasmic regions of CcsB/A proteins vary, although all possess a conserved Beta stranded region (called the Beta cap) that fits the H. hepaticus open conformation electron density.
SuppFig6B. The CcsBA Beta cap primary sequence best-fit comparisons using Tcoffee.

Supp Fig7. Dimensions of the CcmF pore, which the buoy model suggests heme entry
SuppFig 8. A beta cap in CcmF?

Supplemental Table 1. CcsB/A proteins in the present study

Supplemental Video 1. The CcmF-based mechanisms: heme reduction of holoCcmE, CXXCH binding, heme attachment to CXXCH, and release of cyt c (holoCXXCH)

Supp Fig 1. Fused CcsBA machines, where the *ccsB* and *ccsA* genes are fused into a single ORF.

a. CcsBA topology with conserved or semi-conserved residues in red (from Mendez, Lowder et. al.). Sequence is from *Helicobacter hepaticus*, for which cryo-EM structures are discussed. Based on autodock results for the CXXCH substrate, the pocket for the first cysteine (of CXXCH) is formed by residues encircled in green, and for the second cysteine in blue (see Fig 3). A site of natural proteolysis is shown, which results in the fused CcsBA proteins purifying as two polypeptides.

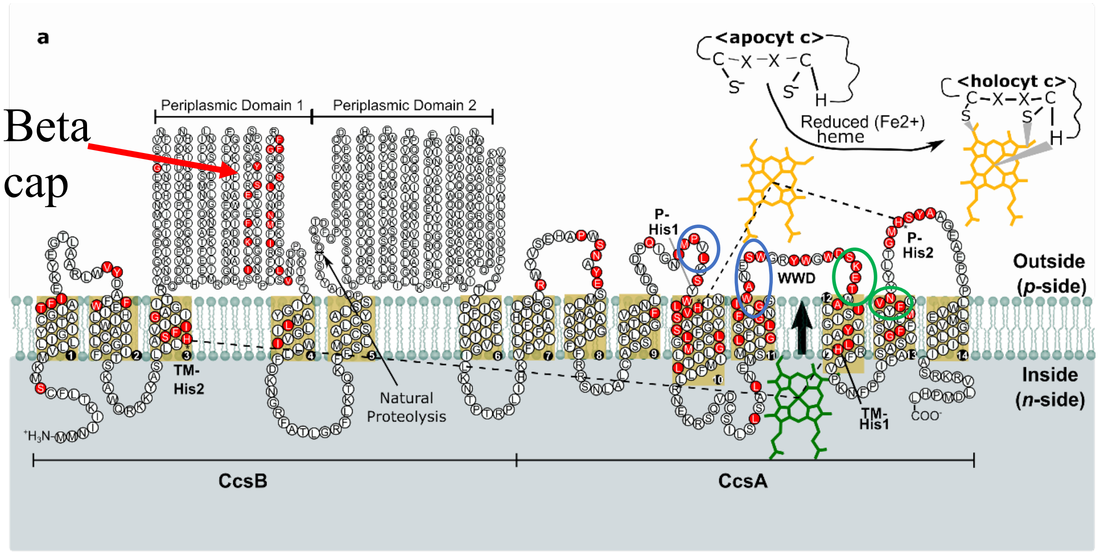

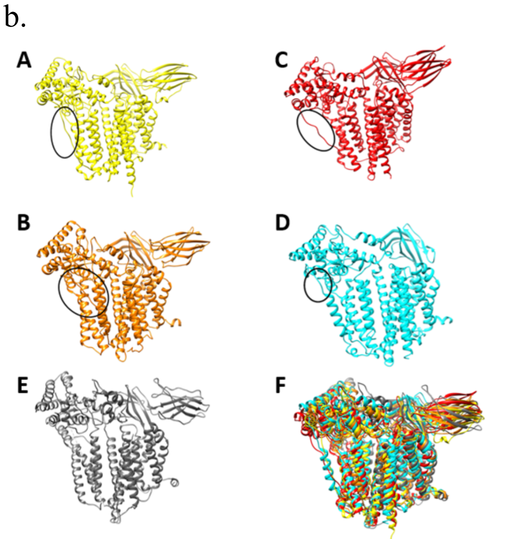

SuppFig1b: Comparison of RosseTTAfold structures of fused CcsBA (A-D) and cryoEM structure (E, open state *H. hepaticus*). The natural proteolysis site is circled in each case and structures are overlayed within the final panel (F). Note that each has the disordered TM region directly after TM5 where the known (for *H. hepaticus*) proteolysis site is located. We speculate that the proteolysis is carried out by the native signal peptidase in the cell, but this requires further study, as does the purpose of the proteolysis. One potential reason is that the proteolysis allows movement to transition from closed to open conformations.

A. NRF1 Wolinella (CcsBA that recognizes CXXCK)
B. CcsA2 Wolinella (CcsBA that recognizes CXXCH)
C. CcsA1 Wolinella (CcsBA that recognizes $CX_{15}CH$)
D. CcsBA *Bacteroides thetamicron*
E. CcsBA *Helicobacter hepaticus* CryoEM open structure
F. Overlay of fused structures

Supp Fig 2. Heme access on the cytoplasmic side to enter the TM-heme binding site A. The cryoEM density of *H.hepaticus* CcsBA is shown from the cytoplasmic side, where parts of the TM-heme are observed, as well as a vestibule where heme could enter. The unfused CcsB-CcsA have a cytoplasmic domain (shown in yellow) not present in the fused proteins. RosseTTAfold analysis places it adjacent to the heme access point, which is fitted from the cryoEM structures of *H.hepaticus* where the heme is displayed. B, The cytoplasmic domain (RoseTTAfold) in unfused CcsA-CcsB, as discussed in the text, potentially for heme access to the TM-heme site. We suggest that possibly this domain provides interactive assistance to ferrochelatase for direct heme insertion. C Protein: protein interaction between CcsB/A and ferrochelatase (*hemH*), as implied by STRING predictions of *H.hepaticus* ferrochelatase (HH_0998 is *ccsBA* using the old locus numbering: new locus ID is WP_041309336).

A

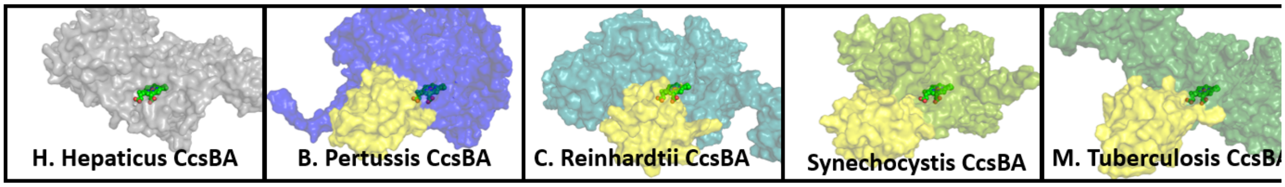

B

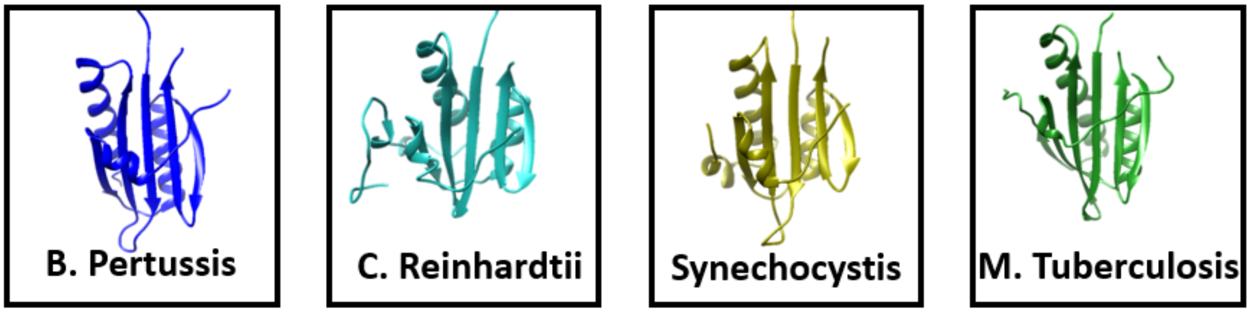

C

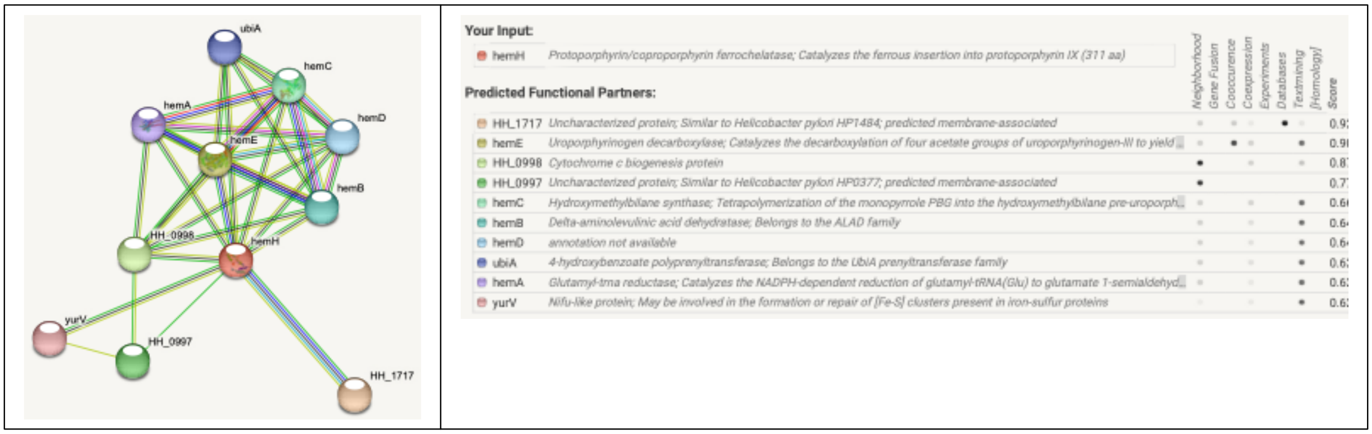

SuppFig 3: The TM-heme binding site in CcsB/A proteins is highly conserved. TM His of CcsBA from various organisms compared to open conformation electron density. The H.hepaticus structure is from the cryoEM open structure with TM-His1 and TM-His2 shown as ligands to the heme. CcsBA RoseTTAFold structures from the indicated organisms shown in colored cartoons and the electron density from *H. hepaticus* open conformation shown in transparent green. TM His ligands from each organism are shown as sticks.

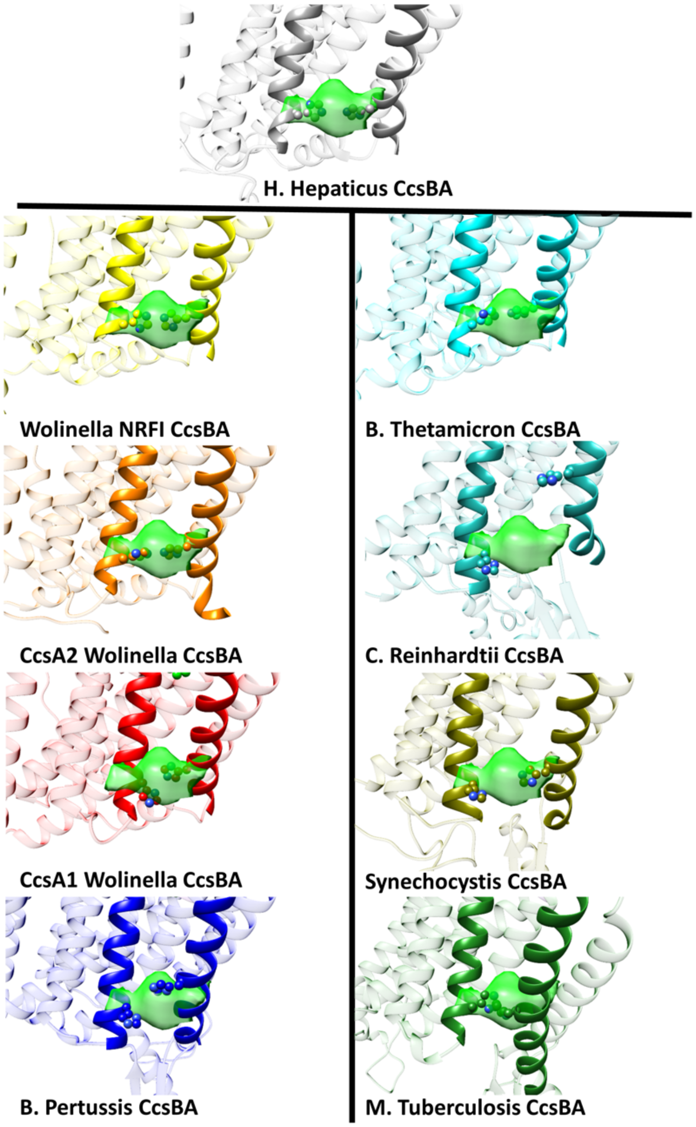

SuppFig 4: Core channel of CcsBA from the indicated organisms is conserved.
The channel of CcsBA including the four core TMs of CcsBA is shown from a top (periplasmic view). In I, the *H. hepaticus* structure of the closed state is used, and the RoseTTAfold structures of the others clearly fit the densities that form the channel. The TM-heme at the bottom of the channel (top view) comes from the *H. hepaticus* densities.

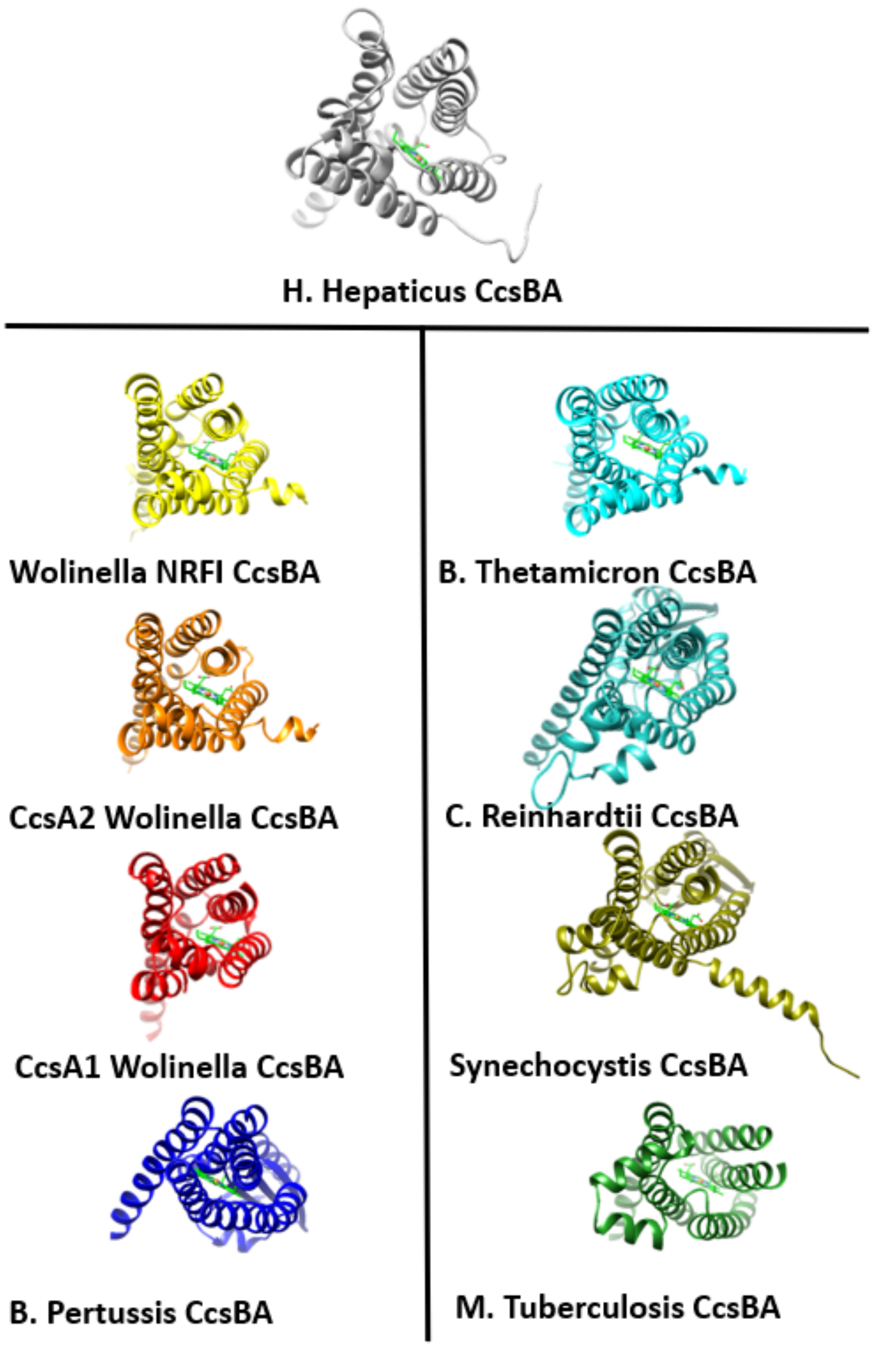

SuppFig 5A: P-His ligands in of CcsBA active site are conserved. The top structure is derived from the *H. hepaticus* cryoEM densities of the open state, with the indicated features. Other structures generated by RoseTTAfold shown with P-His1 in purple and P-His2 in orange. The flexible P-His 2 loop density is shown as an orange cloud. Note that the P-His2 loop is not resolved in the *H. hepaticus* closed state where heme is not present in the P-heme active site, thus it is likely a flexible loop with structure that is stabilized by the heme. Consistent with this, the P-His2 loop is poorly predicted by RosseTTAfold, although the P-His2 ligand is observed near where its *H.hepaticus* counterpart resides.

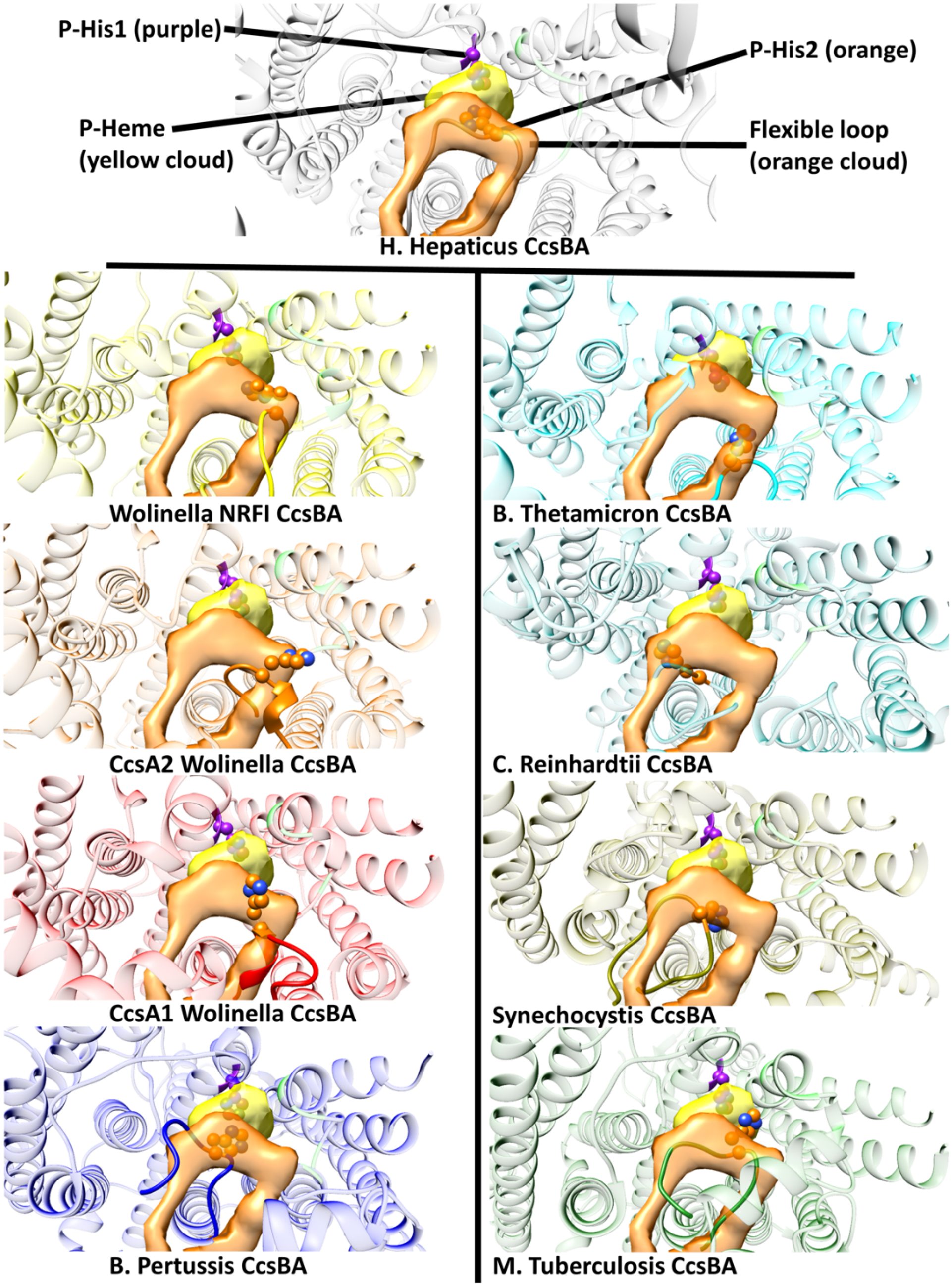

SuppFig 5B: Structures of the WWD domain at the CcsBA active site are conserved. The top structure is derived from the *H. hepaticus* cryoEM densities of the open state, with the indicated features. CcsBA from various organisms. WWD *H. hepaticus* density is shown in green. Shown in sticks in each organism are the four conserved tryptophans, W828, W833, W837, W839 of the WWD domain.

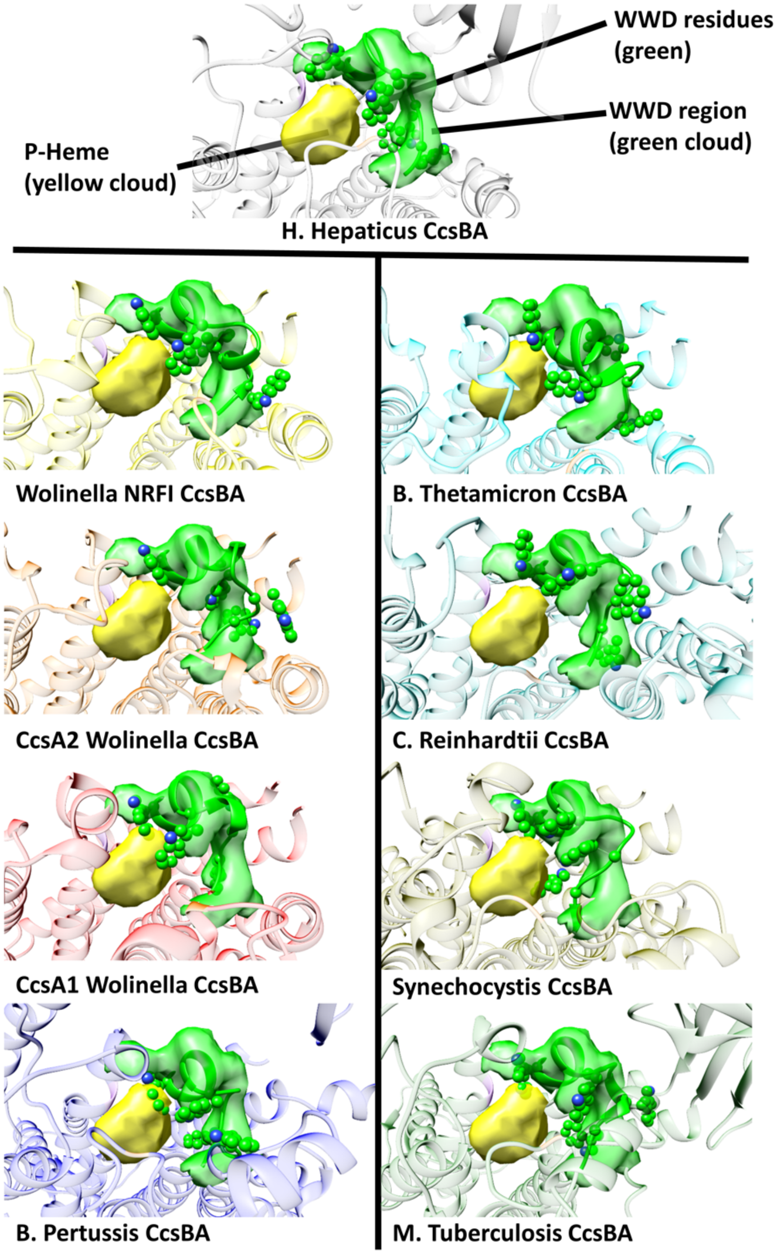

SuppFig 6A: Periplasmic regions of CcsB/A proteins vary, although all possess a conserved Beta stranded region (called the Beta cap) that fits the H. hepaticus open conformation electron density. RoseTTAfold structures of CcsBA from various organisms shown in cartoon with CcsBA open electron density shown in gray with partial transparency. P-Heme from CcsBA open conformation is shown as sticks, and the Betra cap region near the P-heme active site is marked with an oval.

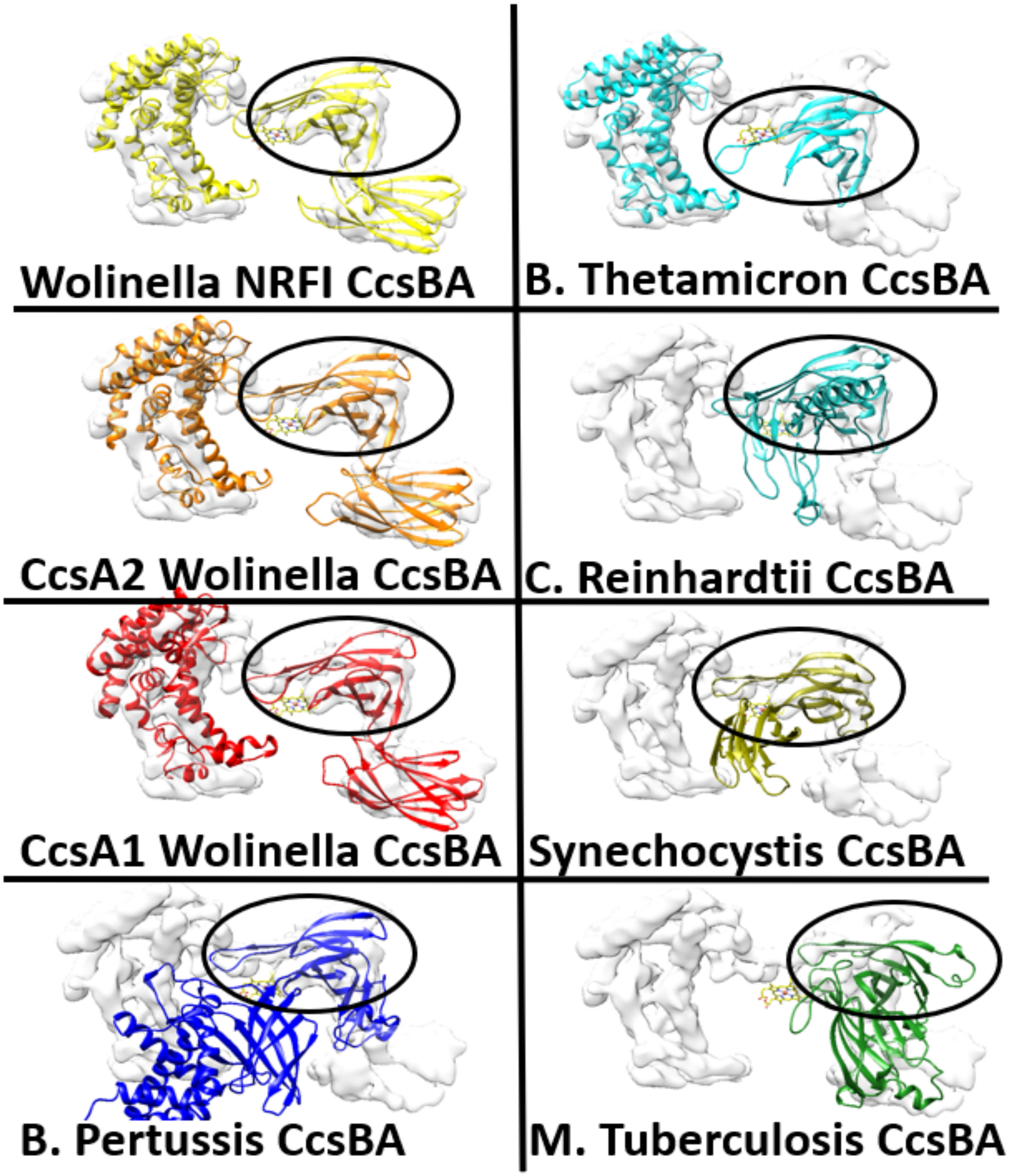

SuppFig6B. The CcsBA Beta cap primary sequence best-fit comparisons using Tcoffee.

```
CcsB Box Sequences                                                                           Flexible loop
H. Hepaticus     -EGLMN-------------------LREGQSVNFI------------NIALPFSIKLKKFELERYPGSNSPASYTSEVEV-LDGQN------PPLPFRIFMNNVLDYGGYRFFQSSYHPDEKGSILSV--
NRFI Wolinella   NEGIMH-------------------IREGESASSF------------EIPLPFSLSLKAFELKRYPGSRSPSEYTSHVAL-VDPSQ-----KVALEETIFMNNTLSYGGYKFFQTSYDMDEKGTILTLN-
CcsA2 Wolinella  FEGTLH-------------------IREGQTQNRM------------EIPLPFSLKLERFELERYPGSMSPSSYASEVIV-IDEGK-----NIEMPYRIYMNHTLDYGGYRFFQSSYDRDEKGTILSVN-
CcsA1 Wolinella  YEGIIH-------------------IREGKSEKEM------------VLSLPFALKLNEFQLERYPGSKSPSSYASEVEV-MTTRG-----ESLFPYRIFMNHPLHFEGYTFFQSSYDLDEKGTVLEVN-
B. Pertussis     -RANLW-------------------VPEGSNASLATIAVND---RSLVQPLPFSLRLKKFTVDYYST-GMPSRFLSLVEV-TDPET-----GEMFDQTIEVNEPLRYKGVTVYQSSFG-----SVFQV--
B. Thetamicron   VTGIMH-------------------IREGETVDRMMRDEG---AGMGNASLPFSVTLDDFRLKRYPGSHSPMSYESDLVI-KK-EN-----EAPLQATVRMNKVIEVDGYRLFQSSFDPDEQGTVLSVSY
C. Reinhardtii   LKGNVM-------------------CPEGQDFQVASFLQPSSPIASMPASASNVIHVNKFTIDYRPD-GSVAQFYSDLSL-LDPAQG---GKEMMRKTISVNDPFRFNGVTMYQTDWSGA---TGLELK-
Synechocystis    FFAQHM-------------------IPSGETFQVSNIIEKG-PLADSQIPKDWGIKVNRFWINYTEN-GAIDQFYSDLSV-VNNQG-----EELDRQTISVNHPLRHRGVTFYQTNWG------------
M. Tuberculosis  YEGNVIVIADGGPGFCSASPAAFDSFRAGNTVDGT-------------SLHPICVRVNNFQAHYLPS-GQATSFAADIDYQADPATADLIANSWRPYRLQVNHPLRV-----------------------
cons               .                         *.  .                     : :. *  .          : : :                    : :*. :
```

Supp Fig7. Dimensions of the CcmF pore, which the buoy model suggests heme entry. CcmF structure (Brausemann et al) van der Waals surface(blue) and CcmF RoseTTAFold van der Waals surface(light blue) shown from the side. The proposed heme entry pore according to the Buoy leaflet model is in yellow. The width of planar heme is approximately 10Å.

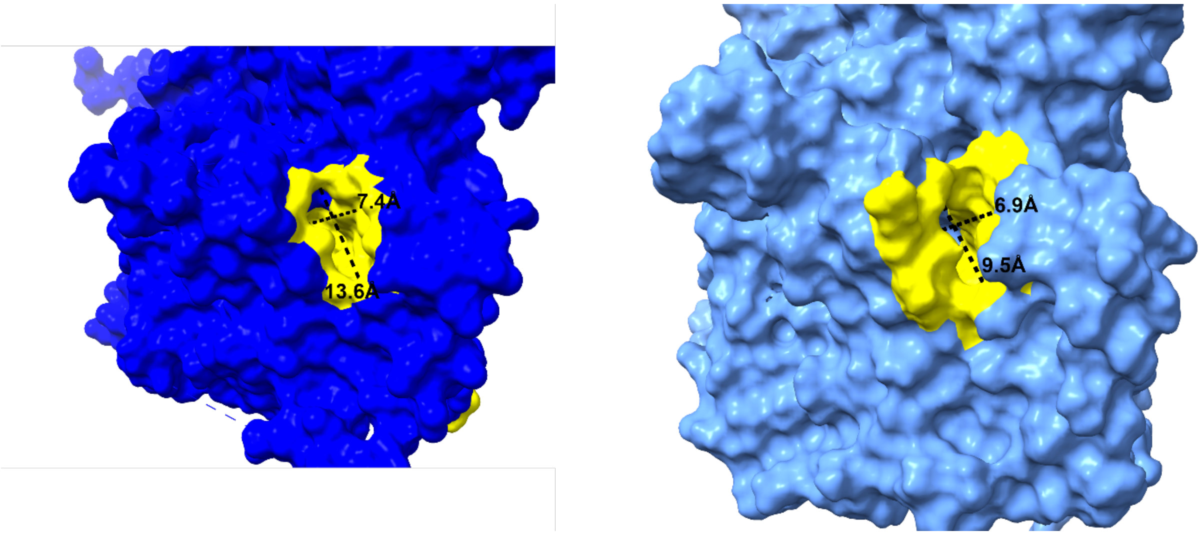

SuppFig 8. A beta cap in CcmF? A. The CcsBA Beta cap structure from H.hepaticus open conformation and B. the periplasmic domain shown by Brausemann et. al.. For "B" the P-heme is positioned according to the periplasmic entry model. C. displays the similar architecture of each region.

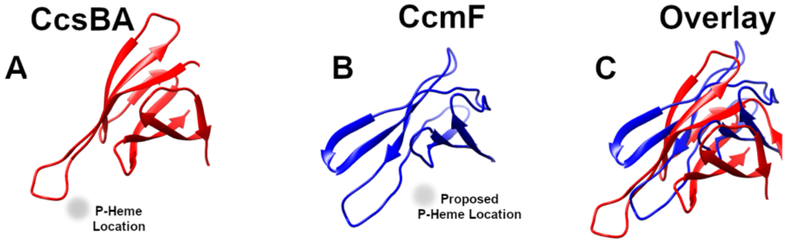

Supplemental Table 1. CcsB/A proteins used in the present study

Organism	Fused (*ccsBA*) or unfused (*ccsB* and *ccsA*)	Presence of cytoplasmic domain.	Number of TMs	Accession numbers	Number of residues in each protein	Comments
Helicobacter hepaticus	fused	No	14	WP_041309336	CcsBA: 936aa	CryoEM densities were used in this study.
Wolinella succinogenes NRFI	fused	No	14	WP_011138865	CcsBA (= NRF1): 902aa	NRF1 is a CcsBA that recognizes a CXXCK motif
Wolinella succinogenes CcsA2	fused	No	14	WP_041571929	CcsBA (=CcsA2): 910aa	CcsA2 is a CcsBA that recognizes a CXXCH motif
Wolinella succinogenes CcsA1	fused	No	14	WP_011138330	CcsBA (=CcsA1): 897aa	CcsA1 is a CcsBA that recognizes a $CX_{15}CH$ motif
Bordetella pertussis	unfused	Yes	17	CcsA: WP_010931573 CcsB: WP_010931572	CcsA: 444aa CcsB: 697aa	No equivalent of *H.hepaticus* TM5 and has extra TM.
Bacteroides thetaiotamicron	fused	No	14	WP_055300122	CcsBA: 811aa	*The B.theta* genome has two CcsBA fused proteins, one of 811 aa and one 686aa.
Chlamydomonas reinhardtii	unfused	Yes	12	CcsA: NP_958384 CcsB: XP_042917413	CcsA: 353 aa CcsB: 614 aa	*ccsA* is chloroplast-encoded while *ccsB* (ccs1) is nuclear encoded, with a chloroplast targeting sequence. No equivalent of *H. hepaticus* TM5 or TM14.
Synechocystis	unfused	Yes	12	CcsA: WP_162327404 CcsB: WP_162329647	CcsA: 334aa CcsB: 458aa	No equivalent of *H. hepaticus* TM5 or TM14
Mycobacterium tuberculosis	unfused	Yes	12	CcsA: WP_003402849 CcsB: WP_003402848	CcsA: 324aa CcsB: 529aa	No equivalent of *H. hepaticus* TM4, TM5 or TM14. Has extra TM.
